# Disparities and Trends in Routine Adult Vaccination Rates Among Disaggregated Asian American Subgroups, National Health Interview Survey 2006–2018

**DOI:** 10.1016/j.focus.2022.100044

**Published:** 2022-10-29

**Authors:** Ziqing Wang, Armaan Jamal, Ryan Wang, Shozen Dan, Shanthi Kappagoda, Gloria Kim, Latha Palaniappan, Jin Long, Jaiveer Singh, Malathi Srinivasan

**Affiliations:** 1 The Stanford Center for Asian Health Research and Education (CARE), Stanford University School of Medicine, Stanford, California; 2 Department of Statistics and Data Science, Cornell University, Ithaca, New York; 3 Department of Medicine, Johns Hopkins University School of Medicine, Baltimore, Maryland; 4 Department of BioSciences, Rice University, Houston, Texas; 5 Department of Computer Science, Rice University, Houston, Texas; 6 Department of Mathematics, Imperial College London, London, United Kingdom; 7 Department of Statistics, Imperial College London, London, United Kingdom; 8 Division of Infectious Diseases & Geographic Medicine, Stanford University School of Medicine, Stanford, California; 9 Division of Cardiovascular Medicine, Stanford University School of Medicine, Stanford, California; 10 Division of Primary Care and Population Health, Department of Medicine, Stanford University School of Medicine, Stanford, California; 11 Department of Pediatrics, Stanford University School of Medicine, Stanford, California; 12 Department of Molecular Biochemistry and Biophysics, Yale University, New Heaven, Connecticut

**Keywords:** Asian American, vaccination, National Health Interview Survey, health disparities, disaggregation

## Abstract

**Introduction:**

Vaccination rates may be improved through culturally tailored messages, but little is known about them among disaggregated Asian American subgroups. We assessed the vaccination rates for key vaccines among these subgroups.

**Methods:**

Using the National Health Interview Survey, we analyzed recent vaccination rates (2015–2018, *n*=188,250) and trends (2006–2018) among Asians (Chinese [*n*=3,165], Asian Indian [*n*=3,525], Filipino [*n*=3,656], other Asian [*n*=5,819]) and non-Hispanic White adults (*n*=172,085) for 6 vaccines (the human papillomavirus, hepatitis B, pneumococcal, influenza, tetanus-diphtheria [tetanus], and shingles vaccines). We controlled demographic, socioeconomic, and health-related variables in multivariable logistic regression and predicted marginal modeling analyses. We also computed vaccination rates among Asian American subgroups on the 2015–2018 National Health Interview Survey data stratified by foreign-born and U.S.-born status. We used Joinpoint regression to analyze trends in vaccination rates. All analyses were conducted in 2021 and 2022.

**Results:**

Among Asians, shingles (29.2%; 95% CI=26.6, 32.0), tetanus (53.7%; 95% CI=51.8, 55.6), and pneumococcal (53.8%; 95% CI=50.1, 57.4) vaccination rates were lower than among non-Hispanic Whites. Influenza (47.9%; 95% CI=46.2, 49.6) and hepatitis B (40.5%; 95% CI=39.0, 42.7) vaccination rates were similar or higher than among non-Hispanic Whites (48.4%; 95% CI=47.9, 48.9 and 30.7%; 95% CI=30.1, 31.3, respectively). Among Asians, we found substantial variations in vaccination rates and trends. For example, Asian Indian women had lower human papillomavirus vaccination rates (12.9%; 95% CI=9.1, 18.0) than all other Asian subgroups (Chinese: 37.9%; 95% CI=31.1, 45.2; Filipinos: 38.7%; 95% CI=29.9, 48.3; other Asians: 30.4%; 95% CI=24.8, 36.7) and non-Hispanic Whites (36.1%; 95% CI=34.8, 37.5). Being male, having lower educational attainment and income, having no health insurance or covered by public health insurance only, and lower frequency of doctor visits were generally associated with lower vaccine uptakes. Foreign-born Asian aggregate had lower vaccination rates than U.S.-born Asian aggregate for all vaccines except for influenza. We also found subgroup-level differences in vaccination rates between foreign-born and U.S.-born Asians. We found that (1) foreign-born Chinese, Asian Indians, and other Asians had lower human papillomavirus and hepatitis B vaccination rates; (2) foreign-born Chinese and Filipinos had lower pneumococcal vaccination rates; (3) foreign-born Chinese and Asian Indians had lower influenza vaccination rates; and (4) all foreign-born Asian subgroups had lower tetanus vaccination rates.

**Conclusions:**

Vaccination rates and trends differed among Asian American subgroups. Culturally tailored messaging and interventions may improve vaccine uptakes.

## HIGHLIGHTS

• Routine vaccination rates in the U.S. are below *Healthy People 2030* goals.
• Asian Americans have lower rates of some routine vaccines than non-Hispanic Whites.
• Routine vaccination rates differ among disaggregated Asian American subgroups.
• Foreign-born Asian Americans have lower vaccination rates than their U.S.-born counterparts.
• Culturally targeted public health interventions may improve vaccination rates.

## INTRODUCTION

Vaccine-preventable diseases cause thousands of hospitalizations and deaths in the U.S. every year.^1^, ^2^, ^3^ Reducing vaccine-preventable diseases and other disparities in preventive healthcare is a major goal of *Healthy People 2030*.^4^ However, racial and ethnic minorities have lower vaccination rates than White Americans.^5^ Although some vaccine-preventable diseases such as hepatitis B disproportionately impact Asian Americans, vaccination rates in the U.S. among Asian Americans are low.^1^^,^^6^^,^^7^

To date, most U.S. vaccination research aggregates data on Asian Americans, despite differences in vaccination rates, health behaviors, and health outcomes between Asian American subgroups.^8^, ^9^, ^10^ Literature on vaccination rates among disaggregated Asian American subgroups is few and far between. One study on influenza vaccination rates has shown large disparities among Asian American subgroups.^11^ One recent study found that most Asian American subgroups have higher coronavirus disease 2019 (COVID-19) vaccination rates than non-Hispanic White (NHW) and other racial/ethnic minority groups, with variations in the Asian subgroups.^12^ However, disaggregated studies on uptakes of other vaccines among Asian Americans are scarce.

Using the National Health Interview Survey (NHIS) from 2006 to 2018, we assessed the rates for 6 routinely recommended vaccines: influenza, pneumococcal (including both polysaccharide and conjugate vaccines), herpes zoster (shingles), hepatitis B, tetanus-diphtheria (tetanus), and human papillomavirus among disaggregated Asian American subgroups and compared rates with those for NHWs. Understanding which groups have higher or lower vaccination rates can inform public health policies and clinical practices to reduce healthcare disparities in the U.S. and help public health practitioners who work in corresponding communities focus on the most relevant interventions.

## METHODS

### Study Sample

The NHIS is a continuous, cross-sectional interview survey that targets the non-institutionalized civilian population in the U.S. Data on sociodemographic information, access to health care, and health behaviors are gathered by trained interviewers from the U.S. Census Bureau. To ensure that the sample is representative of the targeted population, NHIS uses a multistage area probability sampling design with stratification and clustering.^13^^,^^14^ Our study used publicly available NHIS data harmonized by the Integrated Public Use Microdata Service.^15^ We included individuals aged ≥19 years who belong to one of the following racial/ethnic groups: NHW, Chinese, Filipino, Asian Indian, and other Asian (OA). Vaccination rate trends by race were analyzed using data from 2006 to 2018 (*n*=572,961). Racial disparities in vaccination rates were analyzed using pooled data from 2015 to 2018 to achieve relatively up-to-date estimations and sufficient sample sizes in NHWs (*n*=172,085) and Asian subgroups (Chinese: *n*=3,165; Filipino: *n*=3,656; Asian Indian: *n*=3,525; OA: 5,819).

### Measures

We analyzed 6 Centers for Disease Control and Prevention (CDC)-recommended vaccines: the human papillomavirus (HPV), hepatitis B, pneumococcal, influenza, tetanus, and shingles vaccines.^16^ Participants were asked whether they had ever received each vaccine within the recommended time frame. Participants responded to prompts for universal vaccines: *During the past 12 months, have you had a flu vaccination?, Have you received a tetanus shot in the past 10 years?*, and *Have you ever received the hepatitis B vaccine?*. For shingles, participants aged ≥50 years were asked *Have you ever had the Zoster or Shingles vaccine, also called Zostavax®?* Before 2018, participants shared their Zostavax vaccine status only. Starting from 2018, they were asked whether they had either the Zostavax vaccine or the new Shingrix vaccine. Because we used pooled data from 2015 to 2018 and because Zostavax was not routinely recommended for adults aged 50–59 years, we examined the shingles vaccination rates among those aged ≥60 years.^17^ HPV-eligible female adults (aged 12–26 years when vaccine became available in the U.S.) responded to *Have you ever received an HPV shot or vaccine?.*

Race/ethnicity was measured by self-identification. We examined vaccination rates of Asians and NHWs in publicly available NHIS adult data.

Our study included demographic, socioeconomic, and health-related covariates. The demographic variables included age (grouped into 19–26, 27–49, 50–64, and ≥65 years and controlled for in universally recommended vaccines, in unit of year, and controlled for in age-specific vaccines within their respective recommended age ranges), sex (female, male), marital status (currently married, not currently married), and nativity (U.S. born, foreign born and lived ≥10 years in the U.S., foreign born and lived <10 years in the U.S.). Socioeconomic variables included family annual income (≤$34,999, $35,000–$74,999, ≥$75,000), education level (less than high school, high school graduate/GED/some college, bachelor's degree or higher), and type of health insurance coverage (public insurance only, private insurance only, both public and private insurance, and not covered). Health-related covariates included self-reported health status (excellent/very good, good, fair/poor) and the number of visits to a physician's office in the last 12 months (none, 1 visit, 2–3 visits, 4–7 visits, ≥8 visits).

### Statistical Analysis

For each racial/ethnic group, we calculated weighted vaccination rates and their 95% CIs. We used sampling weights stored in the NHIS data set. These weights represent the inverse probability of an observation being selected into the sample, adjusted for nonresponse and complex sampling designs. For each year, these weights sum to the total number of the non-institutionalized civilian population in the U.S. in that year. We computed the weighted rates instead of the raw, unweighted rates to produce nationally representative estimates. We used *t-*tests to compare weighted rates between NHWs and Asian subgroups. Chi-square tests were used to compare the proportions of participants in NHW and Asian subgroups in categories of included population characteristics.

Multivariable logistic regression models were fitted for each vaccination outcome to identify independently associated factors. Wald tests were used to determine the statistical significance of regression coefficients. Predictive marginal models were used to calculate adjusted vaccination rates, which are predictive means of vaccination rates stratified by race/ethnicity while controlling for all covariates in the multivariable logistic regression models. Each adjusted rate was calculated as a probability-weighted average of the fitted probabilities of receiving the vaccine calculated from the regression model over a new, standardized population. In this population, all observations were set to each race/ethnicity, respectively, while holding other covariates at their respective probability-weighted means. We used *t*-tests to compare adjusted vaccination rates between NHWs and Asian subgroups.

Joinpoint, Version 4.9.0.0, was used in 2021 to analyze trends in adult vaccination rates from 2006 to 2018.^18^ The Joinpoint statistical software fits the simplest Joinpoint model allowable by data inputs and uses a Monte Carlo Permutation method to incrementally test for statistically significant joinpoints.^19^ Detecting a Joinpoint means that the model favors the alternative hypothesis that there is a change in trend at the time corresponding to the Joinpoint. Therefore, the software tests the statistical significance of changes in trend and visualizes the fitted models as different line segments connected by joinpoints.^18^^,^^19^ For HPV, shingles, and tetanus vaccines, data from 2008 (instead of 2006) to 2018 were used in Joinpoint analyses because they were unavailable in NHIS until 2008. Two measures were used to characterize trend. The annual percentage change (APC) is the percentage change in vaccination rate compared with that in the previous year within the same time segment (between the starting time point and the first Joinpoint, between 2 consecutive joinpoints, or between the last Joinpoint and the ending time point). The average APC (AAPC) is the weighted average of all APCs, where each weight is the length of the time segment over which each APC was computed.^18^ If no Joinpoint was detected, the 2 measures are equal.

We conducted additional analyses on the 2015–2018 data stratified by U.S.-born versus foreign-born status, where we calculated the weighted and adjusted vaccination rates among U.S.-born Asian subgroups and foreign-born Asian subgroups, separately.

All statistical analyses except Joinpoint regression were performed in 2021 and 2022 using R, Version 4.1.0.^20^ This study was considered not human subject research by Stanford IRB.

## RESULTS

**Figure 1:**
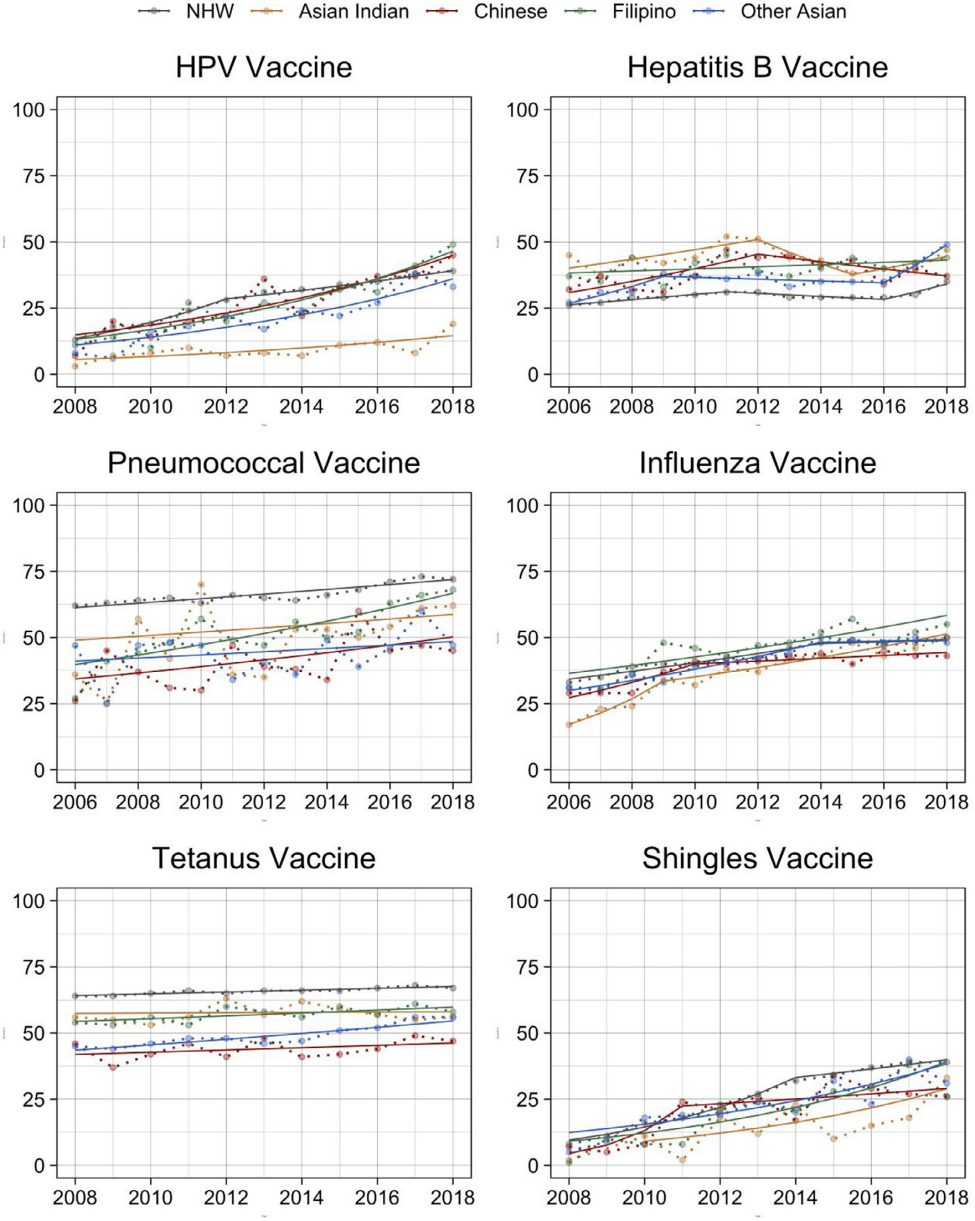
Trends of vaccination rates in Asian Indians, Chinese, Filipinos, other Asians, and NHWs, NHIS.^15^ ^a^Vaccination rates of the HPV vaccine in Asian ethnic subgroups and NHWs, including age-appropriate female adults. ^b^Vaccination rates of the hepatitis B vaccine in Asian ethnic subgroups and NHW adults. ^c^Vaccination rates of the pneumococcal vaccine in Asian ethnic subgroups and NHW adults. ^d^Vaccination rates of the influenza vaccine in Asian ethnic subgroups and NHW adults. ^e^Vaccination rates of the tetanus-diphtheria vaccine in Asian ethnic subgroup and NHW adults. ^f^Vaccination rates of the shingles vaccine in Asian ethnic subgroups and NHWs, including adults aged ≥60 years. ^g^Weighted vaccination rates for each survey year are connected by dotted lines. Fitted Joinpoint regressions are represented in solid lines of different colors that correspond to each race/ethnicity investigated. ^h^To avoid cluttering, 95% CIs for each time point were not included in the above figure. For each vaccine and all races/ethnicities, tables that contain the estimate, the standard error, and the 95% CIs for identified Joinpoints and their corresponding intercepts and slopes are included in the Appendix (available online). ^i^Line segments in the figure and in the Appendix Tables (available online) indicate a statistically significant change in the estimated APC of the vaccine uptake at the year where the new line segment starts. ^j^The start year of the HPV vaccine, the tetanus vaccine, and the shingles vaccine is 2008 instead of 2006 because NHIS first started asking for the vaccination status of these 3 vaccines in 2008. APC, annual percentage change; HPV, human papillomavirus; NHIS, National Health Interview Survey; NHW, non-Hispanic White.

From 2015 to 2018, we included 16,165 Asian respondents (3,165 Chinese, 3,656 Filipinos, 3,525 Asian Indians, and 5,819 OAs). In this population, weighted vaccination rates were 28.4% (95% CI=25.1, 31.8) for HPV vaccines among age-eligible females, 40.5% (95% CI=39.0, 42.2) for hepatitis B vaccines, 53.8% (95% CI=50.1, 57.4) for pneumococcal vaccines among those aged ≥65 years, 47.9% (95% CI=46.2, 49.6) for influenza vaccines, 53.7% (95% CI=51.8, 55.6) for tetanus vaccines, and 29.2% (95% CI=26.6, 32.0) for shingles vaccines among those aged ≥60 years (Table 1). Figure 1 visualizes the vaccination rate trends (Appendix Tables 1-6, available online).

**Table 1:** Characteristics and Unadjusted Vaccination Rates of Asian and NHW Adults Aged >18, NHIS^15^

Characteristics	NHW, % (95% CI)	All Asian,% (95% CI)	Chinese, % (95% CI)	Filipino, % (95% CI)	Asian Indian, % (95% CI)	Other Asian, % (95% CI)
Unadjusted number of participants (total = 188,250)	172,085	16,165	3,165	3,656	3,525	5,819
Sex						
Female	51.5 (51.3, 51.6)	**53.4 (52.8, 54.0)**	**54.7 (53.2, 56.1)**	**56.9 (55.5, 58.3)**	**48.6 (47.4, 49.8)**	**54.0 (52.9, 55.1)**
Age						
19–26	12.2 (11.9, 12.5)	**14.5 (13.7, 15.4)**	**17.3 (15.5, 19.3)**	12.6 (11.3, 14.1)	12.6 (11.3, 14.1)	**15.4 (14.1, 16.7)**
27–49	35.7 (35.3, 36.2)	**47.8 (46.3, 49.2)**	**42.8 (40.4, 45.2)**	**43.0 (41.0, 45.0)**	**61.0 (57.9, 64.1)**	**44.0 (42.1, 46.0)**
50–64	27.9 (27.6, 28.3)	**22.4 (21.5, 23.3)**	**23.7 (21.8, 25.8)**	**23.9 (22.2, 25.7)**	**17.0 (15.2, 19.0)**	**24.5 (23.2, 25.9)**
≥65	24.1 (23.7, 24.5)	**15.3 (14.3, 16.3)**	**16.1 (14.3, 18.2)**	**20.4 (18.7, 22.3)**	**9.3 (8.1, 10.8)**	**16.0 (14.4, 17.8)**
Marital status						
Married	58.6 (58.1, 59.1)	**65.8 (64.6, 66.9)**	**64.4 (62.0, 66.8)**	61.3 (58.9, 63.6)	**75.1 (72.9, 77.2)**	**62.6 (60.8, 64.4)**
Nativity						
U.S. born	94.5 (94.2, 94.8)	**22.3 (21.1, 23.5)**	**19.6 (17.8, 21.6)**	**35.7 (32.9, 38.7)**	**11.2 (9.9, 12.7)**	**23.9 (21.9, 26.1)**
≤10 years in the U.S.	0.89 (0.80, 0.99)	**20.5 (19.1, 21.9)**	**22.2 (19.7, 24.9)**	**12.0 (10.4, 13.8)**	**31.1 (27.8, 34.6)**	**16.9 (15.1, 19.0)**
>10 years in the U.S.	4.5 (4.3, 4.8)	**57.2 (55.8, 58.6)**	**58.2 (55.6, 60.8)**	**52.3 (49.6, 54.9)**	**57.7 (54.4, 60.9)**	**59.2 (57.0, 61.3)**
Education level						
Less than high school	7.1 (6.9, 7.4)	**8.9 (8.2, 9.8)**	**11.2 (9.2, 13.6)**	**5.6 (4.6, 6.7)**	**4.8 (3.9, 6.0)**	**12.7 (11.3, 14.2)**
HS graduate/GED/some college	56.6 (55.9, 57.2)	**36.9 (35.5, 38.2)**	**32.7 (30.3, 35.0)**	**48.3 (45.7, 50.8)**	**22.1 (19.6, 24.8)**	**43.4 (41.6, 45.2)**
Bachelor's degree or higher	36.3 (35.5, 37.0)	**54.2 (52.5, 55.9)**	**56.1 (52.5, 59.7)**	**46.2 (43.4, 49.0)**	**73.1 (69.9, 76.1)**	**44.0 (41.9, 46.1)**
Family income						
<$35,000	22.5 (21.9, 23.0)	22.3 (20.9, 23.8)	**29.4 (26.4, 32.6)**	**17.5 (15.2, 20.0)**	**15.2 (13.0, 17.6)**	**26.2 (23.8. 28.7)**
$35,000–$74,999	29.0 (28.5, 29.5)	**23.7 (22.4, 25.1)**	**20.7 (18.0, 23.7)**	**22.4 (20.0, 25.1)**	**19.8 (17.4, 22.4)**	29.2 (27.1, 31.4)
>$75,000	48.5 (47.7, 49.3)	**54.0 (52.4, 55.9)**	49.9 (45.7, 54.2)	**60.0 (56.9, 63.1)**	**65.1 (61.7, 68.3)**	**44.6 (41.7, 47.5)**
Health insurance status						
Private only	80.6 (80.1, 81.1)	**74.9 (73.3, 76.5)**	**71.9 (67.9, 75.6)**	79.1 (76.8, 81.2)	**84.1 (81.6, 86.3)**	**67.2 (64.7, 69.6)**
Public only	11.7 (11.3, 12.1)	**18.1 (16.7, 19.6)**	**22.4 (19.0, 26.3)**	13.9 (11.9, 16.1)	10.6 (8.9, 12.6)	**23.7 (21.4, 26.1)**
Both private and public	0.53 (0.48, 0.58)	0.28 (0.19, 0.52)	0.29 (0.11, 0.75)	0.33 (0.14, 0.77)	**0.16 (0.06, 0.45)**	0.33 (0.18, 0.63)
Not covered	7.2 (6.9, 7.4)	6.7 (6.0, 7.4)	**5.3 (4.2, 6.8)**	6.7 (5.6, 8.0)	**5.1 (4.1, 6.5)**	**8.8 (7.6, 10.1)**
Self-reported health status						
Excellent/very good	62.7 (62.2, 63.2)	**65.3 (64.1, 66.6)**	63.7 (61.0, 66.4)	64.9 (62.6. 67.2)	**72.5 (70.3, 74.7)**	61.3 (59.3, 63.3)
Good	25.7 (25.3, 26.1)	25.5 (24.4, 26.5)	26.6 (24.1, 29.2)	25.4 (23.5, 27.4)	**21.2 (19.4, 23.1)**	**27.9 (26.3, 29.6)**
Fair/poor	11.6 (11.4, 11.9)	**9.2 (8.5, 10.0)**	9.7 (8.2, 11.4)	**9.7 (8.4, 11.1)**	**6.3 (5.2, 7.6)**	10.8 (9.5, 12.1)
Number of office visits to a healthcare professional in the last 12 months						
No visits	13.7 (13.3, 14.1)	**21.5 (20.1, 22.9)**	**23.2 (20.7, 30.0)**	**16.8 (14.5, 19.3)**	**21.8 (18.8, 25.2)**	**22.8 (20.6, 25.2)**
1 visit	16.6 (16.2, 16.9)	**23.9 (22.6, 25.2)**	**24.5 (21.8, 27.4)**	**22.8 (20.2, 25.6)**	**26.3 (23.7, 29.0)**	**22.1 (20.2, 24.3)**
2–3 visits	27.4 (27.1, 27.8)	26.6 (25.3, 27.9)	25.6 (22.8, 28.7)	28.3 (25.4, 31.4)	28.2 (25.5, 31.2)	24.8 (22.5, 27.4)
4–7 visits	22.1 (21.7, 22.5)	**16.9 (15.7, 18.1)**	**15.0 (12.9, 17.5)**	19.2 (16.6, 22.2)	**15.0 (12.9, 17.5)**	**18.2 (16.2, 20.4)**
8 visits or more	20.2 (19.8, 20.6)	**11.2 (10.3, 12.2)**	**11.6 (9.7, 14.0)**	**12.9 (10.6, 15.6)**	**8.6 (6.9, 10.7)**	**12.0 (10.2, 13.8)**
Vaccination rate						
HPV vaccine	36.1 (34.8, 37.5)	**28.4 (25.1, 31.8)**	37.9 (31.1, 45.2)	38.7 (29.9, 48.3)	**12.9 (9.1, 18.0)**	30.4 (24.8, 36.7)
Hepatitis B vaccine	30.7 (30.1, 31.3)	**40.5 (39.0, 42.2)**	**38.5 (35.2, 41.8)**	**42.3 (39.0, 45.7)**	**41.2 (37.9, 44.5)**	**40.4 (37.9, 43.0)**
Pneumococcal vaccine	71.1 (70.4, 71.9)	**53.8 (50.1, 57.4)**	**48.7 (41.0, 56.5)**	**62.8 (56.9, 68.4)**	**57.7 (48.0, 66.8)**	**48.4 (42.9, 53.8)**
Influenza vaccine	48.4 (47.9, 48.9)	47.9 (46.2, 49.6)	**43.1 (40.1, 46.1)**	**53.0 (49.6, 56.4)**	47.1 (43.8, 50.4)	48.8 (46.1, 51.5)
Tetanus-diphtheria vaccine	67.1 (66.4, 67.7)	**53.7 (51.8, 55.6)**	**45.7 (42.1, 49.4)**	**58.7 (55.2, 62.1)**	**56.6 (53.2, 60.0)**	**53.8 (50.7, 56.9)**
Shingles vaccine	37.6 (36.8, 38.3)	**29.2 (26.6, 32.0)**	**28.7 (22.7, 35.5)**	**30.5 (26.1, 35.4)**	**20.6 (14.6, 28.1)**	**31.8 (27.5, 36.4)**

*Note:* Boldface indicates statistically significant (*p*<0.05 by chi-square tests and *t*-tests for population characteristics and vaccination rates, respectively) difference from NHWs.

NHIS, National Health Interview Survey; NHW, non-Hispanic White.

### Human Papillomavirus Vaccine

Although the estimated HPV vaccination rate for age-eligible adult females was significantly lower among aggregated Asians (28.4%) than among NHWs (36.1%), it is not significantly different among Chinese (37.9%), Filipinos (38.7%), and OAs (30.4%) from that among NHWs. Notably, age-eligible adult Asian Indian females (12.9%) had significantly lower vaccination rates than all included groups (Table 1). Adjusted HPV vaccination rates were 38.4% for Chinese, 44.9% for Filipinos, and 36.1% for OAs, all similar to or higher than that for NHWs (35.6%). However, the adjusted HPV vaccination rate in age-eligible adult Asian Indian females (23.2%) remained the lowest (Table 2).

**Table 2:** Adjusted Vaccination Rates by Race/Ethnicity for Asian and NHW Adults,^a^ NHIS,^15^ per 100 People

Racial/ethnic groups	HPV vaccine, % (95% CI)	Hepatitis B vaccine, % (95% CI)	Pneumococcal vaccine, % (95% CI)	Influenza vaccine, % (95% CI)	Tetanus diphtheria vaccine, % (95% CI)	Shingles vaccine, % (95% CI)
**NHW (*n*=172,085)**	35.6 (34.2, 37.0)	34.0 (33.4, 34.7)	71.8 (70.8, 72.8)	44.5 (43.9, 45.0)	67.6 (66.9, 68.3)	36.8 (35.9, 37.7)
**Chinese (*n*=3,165)**	38.4 (30.8, 45.9)	36.0 (32.8, 39.3)	64.8 (54.8, 74.8)	**49.7 (46.5, 52.9)**	**56.9 (53.2, 60.6)**	34.1 (24.6, 43.5)
**Filipino (*n*=3,656)**	44.9 (34.0, 55.8)	**45.0 (41.4, 48.6)**	73.7 (64.7, 82.6)	**56.8 (53.0, 60.6)**	66.8 (63.1, 70.6)	36.5 (28.3, 44.6)
**Asian Indian (*n*=3,625)**	**23.2 (16.3, 30.2)**	35.6 (32.1, 39.0)	79.8 (71.2, 88.3)	**55.1 (51.8, 58.4)**	66.7 (61.2, 67.6)	26.8 (16.5, 37.2)
**Other Asian (*n*=5,819)**	36.1 (29.0, 43.2)	**42.3 (39.3, 45.3)**	66.8 (60.1, 73.5)	**56.0 (53.1, 58.9)**	64.4 (61.2, 67.6)	**44.4 (37.7, 51.0)**

*Note:* Boldface indicates a statistically significant (*p*<0.05 by *t*-tests) difference from NHWs.

NHIS, National Health Interview Survey; NHW, non-Hispanic White.

a Vaccination rates adjusted for all covariates included in the multivariable logistic regression.

HPV vaccination rates increased for all groups from 2008 to 2018 at similar rates (Appendix Table 7, available online) for Chinese (AAPC=11.7, 95% CI=6.1, 17.5), Filipinos (AAPC=13.5, 95% CI=8.0, 19.2), Asian Indians (AAPC=10.1, 95% CI=2.7, 18.1), OAs (AAPC=12.5, 95% CI=7.2, 18.1), and NHWs (AAPC=11.3, 95% CI=8.3, 14.3).

### Hepatitis B Vaccine

The estimated hepatitis B adult vaccination rates were 38.5% for Chinese, 42.3% for Filipinos, 41.2% for Asian Indians, and 40.4% for OAs, all significantly higher than that for NHWs (30.7%) (Table 1). The adjusted hepatitis B vaccination rates for Chinese (36.0%) and Asian Indians (35.6%) were not significantly different from that for NHWs (34.0%), whereas Filipinos (45.0%) and OAs (42.3%) had higher rates than NHWs (Table 2).

From 2006 to 2018, the hepatitis B vaccination rate remained stable (Appendix Table 9, available online) for Chinese (AAPC=1.6; 95% CI= –1.7, 4.9), Filipinos (AAPC=1.0; 95% CI= –0.7, 2.7), and Asian Indians (AAPC=0.9; 95% CI= –5.4, 7.6). It moderately increased for OAs (AAPC=5.2; 95% CI=1.9, 8.5) and NHWs (AAPC=2.2; 95% CI=0.9, 3.6). Chinese (APC=6.7; 95% CI=0.8, 12.8) and NHW (APC=3.5; 95% CI=1.4, 5.7) had increasing trends in the hepatitis B vaccination rate from 2006 to 2012 and 2011, respectively. OAs (APC=19.1; 95% CI=1.5, 39.6) and NHW (APC=9.7; 95% CI=1.6, 18.5) had increasing trends from 2016 to 2018. Chinese (APC= –3.3; 95% CI= –8.1, 1.8), Asian Indians (APC= –9.5; 95% CI= –31.2, 18.9), OAs (APC= –1.0; 95% CI= –3.5, 1.6), and NHW (APC= –1.9; 95% CI= –4.3, 0.4) had negative, although statistically insignificant, point estimates of APC for hepatitis B vaccination rates from 2006 to 2018, 2015, 2016, and 2016, respectively (Appendix Table 10, available online).

### Pneumococcal Vaccine

The estimated pneumococcal vaccination rates among adults aged ≥65 years were 48.7% for Chinese, 62.8% for Filipinos, 57.7% for Asian Indians, and 48.4% for OAs, all significantly lower than that for NHWs (71.1%) (Table 1). Chinese (64.8%) and OAs (66.8%) had lower point estimates for adjusted pneumococcal vaccination rates than NHWs (71.8%). Filipinos (73.7%) and Asian Indians (79.8%) had higher point estimates for adjusted vaccination rates than NHWs (Table 2). However, these differences were not statistically significant.

From 2006 to 2018, pneumococcal vaccination rates significantly increased for Filipinos (AAPC=4.4; 95% CI=2.5, 6.3) and NHWs (AAPC=1.3; 95% CI=0.9, 1.8) and displayed nonsignificant upward trends for Chinese (AAPC=3.2; 95% CI= –0.4, 6.9), Asian Indians (AAPC=1.5; 95% CI= –2.0, 5.2), and OAs (AAPC=1.4; 95% CI= –1.6, 4.5). No Joinpoint was detected (Appendix Table 11, available online).

### Influenza Vaccine

Although the estimated adult influenza vaccination rate was not statistically significantly different between aggregated Asians (47.9%) and NHWs (48.4%), it is significantly higher in Filipinos (53.0%) and significantly lower in Chinese (43.1%) than in NHWs (Table 1). The adjusted influenza vaccination rates for all Asian subgroups were higher than those for NHWs—49.7% for Chinese, 56.8% for Filipinos, 55.1% for Asian Indians, 56.0% for OAs, and 44.5% for NHWs (Table 2).

Influenza vaccination rates increased for all groups from 2006 to 2018 (Appendix Table 13, available online). Influenza vaccination rates overall increased at a similar or faster pace in Chinese (AAPC=4.2; 95% CI=1.8, 6.6), Filipinos (AAPC=4.0; 95% CI=2.1, 5.9), Asian Indians (AAPC=4.6; 95% CI=4.6, 14.8), and OAs (AAPC=4.2; 95% CI 2.9, 5.6) than in NHWs (AAPC=3.0; 95% CI=2.1, 4.0). However, the influenza vaccination leveled off for several Asian groups (Appendix Table 14, available online). For example, the influenza vaccination rate in Chinese increased from 2006 to 2010 (APC=10.1; 95% CI=2.5, 18.4) but plateaued from 2010 to 2018 (APC=1.3; 95% CI= –0.5, 3.1). For OAs, it increased from 2006 to 2014 (APC=6.1; 95% CI=4.6, 7.7) but subsided from 2014 to 2018 (APC=0.5; 95% CI= –2.9, 4.0).

### Tetanus-Diphtheria (Tetanus) Vaccine

The estimated adult tetanus vaccination rates were 45.7% for Chinese, 58.7% for Filipinos, 56.6% for Asian Indians, and 53.8% for OAs, all significantly lower than those for NHWs (67.1%) (Table 1). Filipinos (66.8%), Asian Indians (66.7%), and OAs (64.4%) had similar adjusted tetanus vaccination rates to those of NHWs (67.6%), whereas Chinese (56.9%) had the lowest rate compared with NHWs (Table 2).

From 2008 to 2018, tetanus vaccination rate moderately increased for Filipinos (AAPC=1.0; 95% CI=0.0, 1.9), OAs (AAPC=2.3; 95% CI=1.4, 3.2), and NHWs (AAPC=0.5; 95% CI=0.3, 0.7). Asian Indians (AAPC=0.1; 95% CI= –1.2, 1.4) and Chinese (AAPC=1.0; 95% CI= –0.8, 2.8) showed nonsignificant upward trends (Appendix Table 15, available online). No Joinpoint was detected (Appendix Table 16, available online).

### Shingles Vaccine

The estimated shingles vaccination rates among adults aged ≥60 years were 28.7% for Chinese, 30.5% for Filipinos, 20.6% for Asian Indians, and 31.8% for OAs, all significantly lower than that for NHWs (37.6%) (Table 1). Although adjusted shingles vaccination rates in Chinese (34.1%), Filipinos (36.5%), and Asian Indians (26.8%) were not significantly different from those in NHWs (36.8%), OAs (44.4%) had significantly higher adjusted rate than NHWs (Table 2).

From 2008 to 2018, the estimated shingles vaccination rates increased for Filipinos (AAPC=15.7; 95% CI=6.6, 25.5), Asian Indians (AAPC=15.5; 95% CI=2.8, 29.7), OAs (AAPC=12.0; 95% CI=6.3, 17.9), and NHWs (AAPC=15.4; 95% CI=12.1, 18.8). Chinese (AAPC=20.6; 95% CI= –0.2, 45.7) showed a nonsignificant upward trend (Appendix Table 17, available online).

### Other Factors Associated With Vaccination

Besides race/ethnicity, we identified other factors independently associated with vaccination rates (Table 3). Generally, being female, having a higher family income, having higher educational levels, having private health insurance, and having higher health-seeking behaviors positively associate with vaccination. Better self-reported health positively associates with HPV, shingles, tetanus, and hepatitis B vaccinations but negatively associates with influenza and pneumococcal vaccinations.

**Table 3:** Multivariable Logistic Regression on Asian and NHW Adult Vaccination Rates, NHIS^15^

Characteristics	HPV vaccine, AOR^a^ (95% CI)	Hepatitis B vaccine, AOR (95% CI)	Pneumococcal vaccine, AOR (95% CI)	Influenza vaccine, AOR (95% CI)	Tetanus-diphtheria vaccine, AOR (95% CI)	Shingles vaccine, AOR (95% CI)
Race						
NHW	Ref	Ref	Ref	Ref	Ref	Ref
All Asian	1.01 (0.78, 1.30)	**1.32 (1.20, 1.45)**	0.93 (0.70, 1.24)	**1.61 (1.45, 1.78)**	**0.84 (0.76, 0.93)**	1.07 (0.84, 1.30)
Chinese	1.15 (0.78, 1.71)	1.10 (0.94, 1.30)	0.70 (0.43, 1.13)	**1.28 (1.10, 1.49)**	**0.62 (0.53, 0.73)**	0.88 (0.56, 1.39)
Filipino	1.60 (0.93, 2.76)	**1.67 (1.42, 1.97)**	1.11 (0.67, 1.84)	**1.78 (1.49, 2.14)**	0.96 (0.81, 1.15)	0.98 (0.67, 1.44)
Asian Indian	**0.49 (0.31, 0.78)**	1.08 (0.91, 1.28)	1.61 (0.90, 2.88)	**1.65 (1.41, 1.93)**	0.96 (0.82, 1.12)	0.61 (0.35, 1.07)
Other Asian	1.03 (0.70, 1.50)	**1.48 (1.29, 1.70)**	0.77 (0.55, 1.09)	**1.72 (1.50, 1.98)**	**0.86 (0.75, 0.99)**	**1.41 (1.04, 1.91)**
Sex						
Female	NA	Ref	Ref	Ref	Ref	Ref
Male	NA	**0.70 (0.67, 0.73)**	**0.83 (0.75, 0.92)**	**0.82 (0.78, 0.85)**	**1.21 (1.16, 1.26)**	**0.75 (0.69, 0.80)**
Age						
19–26	NA	Ref	NA	Ref	Ref	NA
27–49	NA	**0.55 (0.51, 0.60)**	NA	1.03 (0.96, 1.12)	**0.72 (0.67, 0.77)**	NA
50–64	NA	**0.28 (0.26, 0.30)**	NA	**1.57 (1.45, 1.70)**	**0.67 (0.62, 0.72)**	NA
≥65	NA	**0.13 (0.12, 0.14)**	NA	**3.98 (3.66, 4.31)**	**0.49 (0.45, 0.53)**	NA
Within HPV age-eligible adult females	**0.85 (0.84, 0.86)**	NA	NA	NA	NA	NA
Within ≥60 (shingles)	NA	NA	NA	NA	NA	**1.040 (1.037, 1.047)**
Within ≥65 (pneumococcal)	NA	NA	**1.06 (1.05, 1.07)**	NA	NA	NA
Marital status						
Married	Ref	Ref	Ref	Ref	Ref	Ref
Not married	**1.70 (1.50, 1.94)**	0.98 (0.93, 1.03)	**0.87 (0.78, 0.97)**	**0.87 (0.83, 0.91)**	**0.95 (0.91, 0.99)**	**0.88 (0.80, 0.95)**
Nativity						
U.S. born	Ref	Ref	Ref	Ref	Ref	Ref
≤10 years in the U.S.	**0.37 (0.27, 0.51)**	1.04 (0.89, 1.22)	**0.41 (0.18, 0.94)**	**0.60 (0.51, 0.71)**	**0.50 (0.43, 0.58)**	**0.42 (0.19, 0.91)**
>10 years in the U.S.	0.77 (0.57, 1.06)	0.96 (0.86, 1.06)	**0.42 (0.34, 0.53)**	**0.76 (0.69, 0.84)**	**0.60 (0.55, 0.66)**	**0.60 (0.50, 0.72)**
Education level						
Bachelor's or higher	Ref	Ref	Ref	Ref	Ref	Ref
HS graduate/GED/some college	**0.48 (0.42, 0.54)**	**0.71 (0.68, 0.74)**	**0.89 (0.80, 0.99)**	**0.65 (0.62, 0.68)**	**0.88 (0.84, 0.91)**	**0.73 (0.67, 0.79)**
Less than high school	**0.29 (0.21, 0.41)**	**0.43 (0.38, 0.48)**	**0.66 (0.55, 0.78)**	**0.60 (0.55, 0.65)**	**0.72 (0.66, 0.77)**	**0.45 (0.38, 0.53)**
Family income						
>$75,000	Ref	Ref	Ref	Ref	Ref	Ref
$35,000–$74,999	0.86 (0.68, 0.89)	0.95 (0.90, 1.00)	0.94 (0.82, 1.06)	**0.83 (0.79, 0.87)**	**0.93 (0.88, 0.98)**	**0.87 (0.79, 0.96)**
<$35,000	0.78 (0.73, 1.02)	0.96 (0.89, 1.02)	**0.84 (0.72, 0.97)**	**0.85 (0.80, 0.91)**	**0.84 (0.78, 0.90)**	**0.66 (0.59, 0.73)**
Health insurance status						
Private only	Ref	Ref	Ref	Ref	Ref	Ref
Public only	0.91 (0.77, 1.08)	0.93 (0.86, 1.00)	**0.78 (0.66, 0.91)**	**0.85 (0.80, 0.91)**	**0.91 (0.85, 0.97)**	0.91 (0.81, 1.03)
Both private and public	0.96 (0.53, 1.75)	1.19 (0.91, 1.56)	0.98 (0.61, 1.58)	1.21 (0.93, 1.57)	0.96 (0.74, 1.25)	1.31 (0.89, 1.92)
Not covered	0.84 (0.67, 1.05)	**0.80 (0.73, 0.88)**	**0.16 (0.07, 0.36)**	**0.36 (0.33, 0.40)**	**0.76 (0.85, 0.97)**	**0.24 (0.16, 0.37)**
Self-reported health status						
Excellent/very good	Ref	Ref	Ref	Ref	Ref	Ref
Good	0.94 (0.81, 1.08)	**0.95 (0.90, 0.99)**	**1.13 (1.01, 1.26)**	1.04 (0.99, 1.09)	**0.92 (0.88, 0.97)**	**0.77 (0.71, 0.84)**
Fair/poor	0.86 (0.65, 1.14)	**0.87 (0.80, 0.94)**	1.12 (0.97, 1.29)	1.09 (0.97, 1.30)	**0.84 (0.78, 0.91)**	**0.65 (0.57, 0.73)**
Number of visits to doctor's office in the past 12 months						
8 visits or more	Ref	Ref	Ref	Ref	Ref	Ref
4–7 visits	0.97 (0.8, 1.15)	**0.87 (0.81, 0.94)**	**0.77 (0.67, 0.87)**	**0.86 (0.81, 0.91)**	**0.84 (0.78, 0.89)**	1.01 (0.92, 1.11)
2–3 visits	**0.85 (0.73, 0.99)**	**0.84 (0.79, 0.89)**	**0.64 (0.56, 0.73)**	**0.70 (0.66, 0.74)**	**0.75 (0.70, 0.80)**	**0.84 (0.77, 0.92)**
1 visit	**0.66 (0.54, 0.80)**	**0.74 (0.67, 0.80)**	**0.48 (0.40, 0.56)**	**0.52 (0.49, 0.55)**	**0.60 (0.56, 0.65)**	**0.68 (0.59, 0.77)**
No visits	**0.53 (0.42, 0.65)**	**0.59 (0.55, 0.64)**	**0.20 (0.16, 0.24)**	**0.29 (0.27, 0.31)**	**0.40 (0.37, 0.43)**	**0.31 (0.25, 0.37)**

*Note:* Boldface indicates a statistically significant (*p*<0.05 by Wald test) difference from the reference level.

HPV, human papillomavirus; NA, not applicable; NHIS, National Health Interview Survey; NHW, non-Hispanic White.

a Adjusted ORs, adjusted for all variables included in the above table.

We found disparities in vaccination rates between foreign-born and U.S.-born Asians. Foreign-born Asian aggregate had lower vaccination rates than U.S.-born Asian aggregate for all included vaccines except for influenza (Table 4). All foreign-born Asian subgroups, except Filipinos, had significantly lower HPV and hepatitis B vaccination rates than their U.S.-born counterparts. Foreign-born Chinese and Filipinos had significantly lower pneumococcal vaccination rates than their respective U.S.-born counterparts. Foreign-born Chinese and Asian Indians had lower influenza vaccination rates than their respective U.S.-born counterparts. Foreign-born individuals in all Asian subgroups had significantly lower tetanus vaccination rates than U.S.-born individuals in their corresponding subgroups. For the shingles vaccination, only foreign-born OAs had a statistically significantly lower vaccination rate than U.S.-born OAs (Table 4).

**Table 4:** Unadjusted Vaccination Rates by Race/Ethnicity and U.S.-Born/Foreign-Born Status, NHIS^15^

Racial/ethnic groups, by nativity status		HPV vaccine % (95% CI)	Hepatitis B vaccine % (95% CI)	Pneumococcal vaccine % (95% CI)	Influenza vaccine % (95% CI)	Tetanus Diphtheria vaccine % (95% CI)	Shingles vaccine % (95% CI)
NHW (*n*=172,085)	**Ref**	36.1 (34.8, 37.5)	30.7 (30.1, 31.3)	71.1 (70.4, 71.9)	48.4 (47.9, 48.9)	67.1 (66.4, 67.7)	37.6 (36.8, 38.3)
All Asian (*n*=16,165)	**Foreign born (*n*=12,153)**	**21.1 (17.7, 24.9)**	**38.1 (36.2, 40.0)**	**50.0 (45.7, 54.2)**	46.5 (44.6, 48.3)	**49.0 (47.1, 51.0)**	**24.8 (22.1, 27.6)**
	**U.S. born (*n*=3,907)**	**43.6 (37.7, 49.7)**	**48.5 (45.5, 51.6)**	67.6 (60.8, 73.7)	**52.7 (49.6, 55.8)**	68.8 (65.6, 71.8)	**45.4 (38.7, 52.3)**
Chinese (*n*=3,165)	**Foreign born (*n*=2,527)**	31.0 (23.9, 39.2)	34.7 (31.0, 38.6)	**42.5 (34.3, 51.1)**	**40.2 (36.5, 43.6)**	**38.3 (34.3, 42.4)**	**24.9 (19.3, 31.4)**
	**U.S. born (*n*=610)**	**53.5 (39.0, 67.4)**	**51.9 (44.7, 58.9)**	81.6 (64.4, 91.5)	54.5 (47.7, 61.1)	73.8 (67.7, 79.0)	48.4 (31.3, 65.9)
Filipino (*n*=3,656)	**Foreign born (*n*=2,339)**	32.9 (21.6, 46.5)	**42.3 (38.3, 46.4)**	**59.8 (51.8, 67.3)**	**54.5 (50.0, 59.0)**	**54.7 (50.3, 58.9)**	**27.0 (21.6, 32.2)**
	**U.S. born (*n*=1,302)**	44.5 (32.4, 57.4)	**42.4 (36.8, 48.1)**	69.4 (57.7, 79.1)	50.7 (45.2, 56.2)	65.4 (59.9, 70.1)	37.8 (29.0, 47.5)
Asian Indian (*n*=3,625)	**Foreign born (*n*=3,139)**	**9.3 (5.8, 14.6)**	**39.0 (35.6, 42.5)**	**57.6 (47.7, 67.0)**	45.9 (42.6, 49.2)	**54.7 (51.2, 58.1)**	**19.6 (13.6, 27.4)**
	**U.S. born (*n*=367)**	33.1 (20.3, 49.2)	**64.3 (54.0, 73.5)**	60.0 (12.7, 93.9)	**59.2 (49.4, 68.3)**	75.7 (66.0, 83.4)	50.0 (13.1, 86.9)
Other Asian (*n*=5,819)	**Foreign born (*n*=4,148)**	**22.4 (16.2, 30.1)**	**37.4 (34.4, 40.5)**	**44.4 (38.2, 50.9)**	47.6 (44.5, 50.8)	**48.4 (45.1, 51.7)**	**25.6 (21.1, 30.6)**
	**U.S. born (*n*=1,628)**	40.9 (31.4, 51.2)	**47.8 (43.3, 52.3)**	**61.0 (50.8, 70.2)**	51.6 (47.0, 56.2)	67.3 (62.2, 72.0)	**52.1 (42.1, 61.9)**

*Note:* Boldface indicates a statistically significant (*p*<0.05) difference from the reference (NHW) level by chi-square test.

The estimated unadjusted pneumonia vaccination rate for U.S.-born Asian Indians has wide 95% CI owing to small sample size (only 4 Asian Indians in the sample are U.S. born, aged over 65 years, and indicated whether they have received the pneumonia vaccine). The estimated unadjusted shingles vaccination rate for U.S.-born Asian Indians has wide 95% CI owing to small sample size (only 5 Asian Indians in the sample are U.S. born, aged over 60 years, and indicated whether they have received the shingles vaccine).

HPV, human papillomavirus; NA, not applicable; NHIS, National Health Interview Survey; NHW, non-Hispanic White.

After adjusting for covariates, disparities among foreign-born and U.S.-born Asian subgroups remained. For example, foreign-born Asian Indians still had significantly lower HPV vaccination rates than U.S.-born Asian Indians. Foreign-born Chinese and Asian Indians had significantly lower hepatitis B and influenza vaccination rates than their respective U.S.-born counterparts. All foreign-born Asian subgroups had significantly lower tetanus vaccination rates than their respective U.S.-born subgroups. (Table 5).

**Table 5:** Adjusted Vaccination Rates by Race/Ethnicity for Asian and NHW Adults by U.S.-Born/Foreign-Born Status, NHIS,^15^ per 100 People

Racial/ethnic groups, by nativity status		HPV vaccine, % (95% CI)	Hepatitis B vaccine, % (95% CI)	Pneumococcal vaccine, % (95% CI)	Influenza vaccine, % (95% CI)	Tetanus-diphtheria vaccine, % (95% CI)	Shingles vaccine, % (95% CI)
NHW (all)	**Ref (*n*=172,085)**	35.6 (34.2, 37.0)	34.0 (33.4, 34.7)	71.8 (70.8, 72.8)	44.5 (43.9, 45.0)	67.6 (66.9, 68.3)	36.8 (35.9, 37.7)
Chinese (*n*=3,165)	**Foreign born (*n*=2,527)**	**25.3 (18.6, 32.0)**	33.2 (29.5, 36.9)	**46.1 (33.9, 58.3)**	**41.1 (37.8, 44.3)**	**40.9 (36.7, 45.1)**	**24.0 (15.7, 32.3)**
	**U.S. born (*n*=610)**	41.5 (27.9, 55.1)	**44.4 (37.8, 51.1)**	74.5 (54.6, 94.3)	**57.7 (51.4, 64.0)**	72.7 (65.9, 79.5)	37.4 (16.5, 58.4)
Filipino (*n*=3,656)	**Foreign born (*n*=2,339)**	36.6 (22.0, 51.2)	**45.0 (40.4, 49.6)**	64.3 (52.8, 75.8)	**53.4 (48.5, 58.4)**	**57.3 (52.6, 61.9)**	**25.1 (16.6, 33.5)**
	**U.S. born (*n*=1,302)**	44.8 (31.0, 58.5)	**43.6 (38.0, 49.2)**	60.0 (44.2, 75.8)	**53.6 (48.0, 59.2)**	66.0 (60.1, 71.9)	41.0 (27.4, 54.5)
Asian Indian (*n*=3,625)	**Foreign born (*n*=3,139)**	**12.7 (7.4, 18.1))**	33.6 (30.3, 37.0)	66.0 (54.7, 77.3)	47.3 (44.4, 50.2)	**54.2 (50.6, 57.8)**	**19.2 (11.2, 27.2)**
	**U.S. born (*n*=367)**	32.3 (18.8, 45.9)	**51.3 (40.9, 61.6)**	NA	**64.2 (55.4, 73.0)**	75.5 (66.2, 71.9)	NA
Other Asian (*n*=5,819)	**Foreign born (*n*=4,148)**	26.6 (18.6, 34.6)	**41.2 (37.7, 44.7)**	**49.7 (41.2, 58.1)**	**50.8 (47.5, 54.1)**	**52.4 (48.7, 56.2)**	30.6 (23.8, 37.5)
	**U.S. born (*n*=1,628)**	36.1 (26.4, 45.9)	**42.3 (38.6, 47.9)**	69.0 (57.7, 80.2)	**54.8 (50.4, 59.2)**	67.5 (62.3, 72.8)	**53.4 (42.6, 64.1)**

*Note:* Boldface indicates a statistically significant (*p*<0.05) difference from the aggregated NHW population by chi-square test.

The estimated adjusted pneumonia vaccination rate (%) for U.S.-born Asian Indians was inaccurate (99.99; 95% CI=99.97, 100.00) owing to a small sample size (only 4 Asian Indians in the sample are U.S. born, aged over 65 years, and indicated whether they had received the pneumonia vaccine). The estimated adjusted shingles vaccination rate for U.S.-born Asian Indians was inaccurate (2.38e-5; 95% CI= –1.31e-5, 6.08e-5) owing to the small sample size (only 5 Asian Indians in the sample are U.S. born, aged over 60 years, and indicated whether they have received the shingles vaccine).

HPV, human papillomavirus; NA, not applicable; NHIS, National Health Interview Survey; NHW, non-Hispanic White.

Finally, we note that the factors associated with vaccination rates in the stratified data are the same as those identified from the unstratified data. The direction of associations did not change either (Appendix Table 20, available online).

## DISCUSSION

Our study is the first to examine disparities in vaccination rates and trends of 6 CDC-recommended routine adult vaccines among disaggregated Asian subgroups. We found that aggregated Asians had lower adult vaccination rates for tetanus, shingles, and pneumococcal vaccines than NHWs by 20%–24% (in percentage relative difference). We also found differences in vaccination rates among disaggregated Asian subgroups. Asian Indian women had less than half the HPV vaccination rates than Chinese, Filipinos, and NHWs. All Asian subgroups had higher hepatitis B vaccination rates (by ∼30%) than NHWs. Filipinos and OAs had higher or similar influenza vaccination rates to those for NHWs (by 9.5% and 0.01%, respectively), whereas Chinese and Indians had lower influenza vaccination rates than NHWs (by 10.9% and 2.7%, respectively).

The low pneumococcal, shingles, and tetanus vaccination rates among Asian Americans reflect gaps in preventive health care.^21^^,^^22^ There may also be patient- and national-level determinants that result in varying vaccination rates within each subgroup. For instance, cultural factors such as perceived susceptibility, perceived relationship between HPV vaccine and sexual activity, stigma, lack of parent–child communication, and recommendation from countries of origin may impact HPV vaccination rates in Asian Indian Americans.^23^ Specifically, the lower HPV vaccination rate in Asian Indian women may relate to family influence because Asian Indian parents are less likely to encourage their daughters to get the HPV vaccine owing to stigma against sexual activities.^24^^,^^25^ In addition, previous research has shown that Asian Indians are not as engaged in preventive healthcare as OA subgroups.^26^ Acculturation and generation status can also influence HPV vaccine uptakes because studies have shown that first-generation immigrants are less accepting of the HPV vaccine than second-generation immigrants.^27^ Acculturation may also influence attitudes toward preventive care because in Asia, medical care is provided more often for acute episodic illness or chronic conditions, without as much emphasis on preventive care.^28^

For the hepatitis B vaccine, we found a discrepancy between the vaccination rate and the risk of disease related to hepatitis B in the Asian American community. Specifically, we found that despite the hepatitis B vaccination rate being higher in Asian subgroups than in NHWs, Asian Americans also have higher risks of dying from hepatitis B‒related liver cancers.^29^^,^^30^ For instance, Chinese Americans are 6 times more likely to die from liver cancer than NHWs despite a higher hepatitis B vaccination rate.^29^ The discrepancy between hepatitis B vaccination rate and its associated cancer mortality could be explained by low hepatitis B‒related cancer screenings in the Asian communities and the stigma against the hepatitis B infection, which prevents Asians from seeking corresponding preventive care.^31^^,^^32^

Interestingly, we found that within the Asian subgroups, Filipinos' vaccination rates are consistently the highest. Although the precise reasons are unknown, this may be owing to a larger proportion of healthcare workers among the Filipino population in the U.S. and a high proportion of Filipino households comprising healthcare workers. It was estimated that approximately 1 in 4 Filipino working adults are frontline healthcare workers and that 38% of Filipinos live in multigenerational households that include at least 1 healthcare worker. This percentage is higher than that of Asian Americans as a whole (18%).^33^ Future research is needed to elucidate the mechanisms driving the different vaccination rates among Asian American subgroups.

Overall, vaccination rates for all racial/ethnic groups improved over the decade but have plateaued in recent years. We observed higher increases in influenza vaccination rates for all Asian groups than for NHWs, driven in part by the relatively low initial vaccination rates and successful community engagement campaigns that specifically targeted minority groups.^34^ A notable exception was the HPV vaccination rate in Asian Indian women, which rose more slowly than in OA subgroups. We also found that hepatitis B vaccination rates for Asian Indians, Chinese, and Filipinos increased more slowly than for NHWs. Another exception to the overall increasing vaccination trend is that the point estimate for the APC of hepatitis B vaccination rate decreased more than onefold for Chinese from 2012 to 2018 than from 2006 to 2012, suggesting that increasing the hepatitis B vaccination rate in the Chinese population is particularly imperative.

Beyond ethnicity, we found several health behaviors and social demographic factors associated with vaccination rates. For example, higher health-seeking behavior, such as more visits to a doctor's office, was associated with higher vaccination rates. One explanation is that people are more likely to receive doctors’ recommendations to get CDC-recommended vaccines during visits. Having health insurance was also associated with higher vaccination uptake.^35^ Types of health insurance also played a role because we found that people who only had public insurance were significantly less likely to get vaccinated than those who had private insurance for pneumococcal, influenza, and tetanus vaccines. The reason behind different vaccination rates by types of health insurance is not well understood. One potential explanation is the impact of the Affordable Care Act (ACA) on some of the public health insurances such as Medicaid. Specifically, only newly eligible beneficiaries and those who already possess private health insurance enjoy the vaccination benefits unified by the ACA, with only minor exceptions. In addition, the ACA mandates for free vaccination coverage did not extend to traditional Medicaid recipients.^36^ Moreover, one study found that general internists and family physicians had the highest dissatisfaction rate with Medicaid and Medicare PART B than with other insurance plans. More than 50% of the interviewed general internists and family physicians reported a lack of awareness of the ACA's vaccine-specific provisions in Medicaid insurance. Therefore, they are more likely to decide against recommending vaccines to a patient who only has Medicaid or Medicare coverage because of coverage or cost-related concerns.^37^ Future research on how the type of health insurance affects vaccination is needed.

Importantly, we identified that nativity as an influential factor in adult vaccination rates among Asian Americans because U.S.-born Asians generally have significantly higher vaccination rates than their foreign-born counterparts. This gap may be partly explained by foreign vaccine policies that affect the large foreign-born Asian populations in the U.S. For instance, the low shingles vaccination rate among Asian Indians may relate to the fact that the Zostavax vaccine was introduced in India in 2016 but was licensed in the U.S. in 2006. This delayed introduction of the Zostavax vaccine in India may have resulted in a lower vaccination rate among Asian Indian immigrants. Although the HPV vaccine was licensed for use by the Drug Controller General of India since 2008, the vaccine was not included in routine government immunization programs until 2016, when some Indian provinces began initiating public HPV vaccination programs.^38^ Immigration may affect vaccine uptakes among foreign-born Asian subgroups as well because immigrants may have many priorities other than immunizations, difficulties navigating the U.S. healthcare system, and language and cultural barriers in understanding and implementing the U.S. vaccination recommendations.^39^ For example, because the tetanus vaccine is used as a booster for the tetanus‒diphtheria‒pertussis vaccine in the U.S., foreign-born Asian immigrants might put a lower priority on this additional booster and focus on other competing priorities instead. To the best of our knowledge, there is no study on why foreign-born Asians in the U.S. have lower tetanus vaccination rates than their U.S.-born counterparts. Future studies on immigrants’ knowledge, attitudes, and behaviors toward tetanus vaccines are needed.

### Limitations

NHIS is a self-reported, cross-sectional survey. Responses are subject to recall bias and social desirability bias. Although NHIS intended to oversample minority groups, Asian Americans were undersampled relative to other groups and might not accurately represent the Asian American population.^40^ NHIS did not explore why people obtained or did not obtain vaccinations, limiting possibilities of causal inference. Misclassification of race/ethnicity may cause nonrepresentative counts in our data set. NHIS did not include vaccination tradenames used in foreign countries, which may cause information bias, especially for immigrant participants. Finally, because NHIS is conducted in English and Spanish only, non‒English- or ‒Spanish-speaking Asian Americans might be excluded.

## CONCLUSIONS

Our study is the first to present disaggregated vaccination rates and trends among Asian American subgroups. Adult vaccination rates in the U.S. remain below public health goals.^4^ For Asian Americans, especially those who are foreign born, pneumococcal, shingles, HPV, tetanus, and hepatitis B vaccination rates are especially concerning. Public health interventions should target specific contributors to low vaccination rates, with attention to cultural barriers to preventive care.^41^ For example, campaigns to bring awareness around the high prevalence, consequences, and prevention of the hepatitis B virus in a culturally sensitive manner may improve stagnating hepatitis B vaccination rates in the Asian communities.^30^ Similar interventions could focus on Asian Indians around the benefits of the HPV vaccine by addressing cultural biases around preventive care and stigma against sexually transmitted infections. Finally, we note the importance of surveilling trends of vaccination rates to identify any plateau or decrease and act promptly.

## Appendix Supplementary materials

Image, application 1

## Footnotes

Supplementary material associated with this article can be found in the online version at doi:10.1016/j.focus.2022.100044.
