# Supplementary material for: Disparities and Trends in Routine Adult Vaccination Rates Among Disaggregated Asian American Subgroups, National Health Interview Survey 2006–2018
Source: AJPM Focus. 2022 Oct 29;2(1):100044. doi: 10.1016/j.focus.2022.100044 (PMC10546520; doi:10.1016/j.focus.2022.100044)
Supplement: Supplementary file 1 [file mmc1.docx]

**Appendix**

**Appendix Table 1: Parameter estimates of the selected joinpoint model for HPV vaccination**

| Race/Ethnicity | Joinpoint (95% CI) | Intercept Estimate | Intercept Standard Error | Slope Estimate | Slope Standard Error | Slope Change Estimate |
| --- | --- | --- | --- | --- | --- | --- |
| Asian Indian | NA | -**196.591806** | 62.35192 | **0.096465** | 0.030951 | NA |
| Chinese | NA | **-223.912342** | 45.539117 | **0.110561** | 0.022602 | NA |
| Filipinos | NA | **-256.139426** | 43.768957 | **0.126548** | 0.021723 | NA |
| NHW | 2012 (2010, 2013) | **-107.771483** | 17.020334 | **0.05294** | 0.008444 | **-0.134255** |
| Other Asian | NA | **-239.259949** | 43.128169 | **0.118057** | 0.02141 | NA |

Note: Boldface indicates statistical significance (p<0.05) compared to 0

**Appendix Table 2: Parameter estimates of the selected joinpoint model for hepatitis B vaccination**

| Race/Ethnicity | Joinpoint (95% CI) | Intercept Estimate | Intercept Standard Error | Slope Estimate | Slope Standard Error | Slope Change Estimate | Slope Change Standard Error |
| --- | --- | --- | --- | --- | --- | --- | --- |
| Asian Indian | NA | -81.045351 | 40.947499 | 0.039946 | 0.02038 | NA | NA |
| Asian Indian | 2012 (2008, 2013) | 201.094057 | 214.091238 | -0.100283 | 0.106329 | -0.140228 | 0.108265 |
| Asian Indian | 2015 (2011, 2016) | -112.03197 | 130.147016 | 0.055115 | 0.064524 | 0.155398 | 0.124375 |
| Chinese | NA | **-130.386878** | 49.018727 | **0.064412** | 0.024401 | NA | NA |
| Chinese | 2012 | 66.028401 | 44.811446 | -0.03321 | 0.022237 | **-0.097622** | 0.033013 |
| Filipino | NA | -20.988925 | 15.365656 | 0.009984 | 0.007636 | NA | NA |
| NHW | NA | **-70.649873** | 15.950269 | **0.034553** | 0.007942 | NA | NA |
| NHW | 2011 | 38.407191 | 18.773426 | -0.019678 | 0.009324 | **-0.05423** | 0.012248 |
| NHW | 2016 | **-188.686068** | 60.205554 | **0.092968** | 0.029841 | **0.112645** | 0.031264 |
| Other Asian | NA | -216.049752 | 88.393496 | 0.107045 | 0.044042 | NA | NA |
| Other Asian | 2009 | 18.36963 | 20.115662 | -0.009639 | 0.009996 | **-0.116685** | 0.045162 |
| Other Asian | 2016 | -352.82683 | 125.100065 | **0.174486** | 0.062005 | **0.184125** | 0.062806 |

Note: Boldface indicates statistical significance (p<0.05) compared to 0

**Appendix Table 3: Parameter estimates of the selected joinpoint model for pneumococcal vaccination**

| Race/Ethnicity | Joinpoint | Intercept Estimate | Intercept Standard Error | Slope Estimate | Slope Standard Error |
| --- | --- | --- | --- | --- | --- |
| Asian Indian | NA | -31.122846 | 32.238525 | 0.015159 | 0.01601 |
| Chinese | NA | -64.514307 | 32.358509 | 0.031628 | 0.016075 |
| Filipino | NA | **-87.366427** | 16.523761 | **0.043093** | 0.008205 |
| NHW | NA | **-27.096966** | 3.989414 | **0.013264** | 0.001982 |
| Other Asian | NA | -28.669274 | 27.150772 | 0.013848 | 0.013491 |

Note: Boldface indicates statistical significance (p<0.05) compared to 0

**Appendix Table 4: Parameter estimates of the selected joinpoint model for influenza vaccination**

| Race/Ethnicity | Joinpoint (95% CI) | Intercept Estimate | Intercept Standard Error | Slope Estimate | Slope Standard Error | Slope Change Estimate | Slope Change Standard Error |
| --- | --- | --- | --- | --- | --- | --- | --- |
| Asian Indian | NA | **-450.486616** | 183.742915 | **0.22369** | 0.091543 | NA | NA |
| Asian Indian | 2009 (2008, 2016) | **-96.370396** | 16.900068 | **0.047425** | 0.00839 | -0.176265 | 0.091927 |
| Chinese | NA | **-194.821714** | 62.701439 | **0.096471** | 0.03123 | NA | NA |
| Chinese | 2010 (2008, 2014) | -26.774777 | 15.61089 | 0.012865 | 0.00775 | **-0.083605** | 0.032177 |
| Filipino | NA | **-79.680672** | 16.541825 | **0.039218** | 0.008218 | NA | NA |
| NHW | NA | **-84.006541** | 9.595783 | **0.041344** | 0.004773 | NA | NA |
| NHW | 2014 (2012, 2016) | -12.940478 | 21.030994 | 0.006058 | 0.010429 | **-0.035286** | 0.01147 |
| Other Asian | NA | **-120.793177** | 12.902233 | **0.059615** | 0.006418 | NA | NA |
| Other Asian | 2014 (2013, 2016) | -10.225511 | 29.910504 | 0.004715 | 0.014833 | **-0.0549** | 0.016162 |

Note: Boldface indicates statistical significance (p<0.05) compared to 0

**Appendix Table 5: Parameter estimates of the selected joinpoint model for tetanus diphtheria** **vaccination**

| Race/Ethnicity | Joinpoint | Intercept Estimate | Intercept Standard Error | Slope Estimate | Slope Standard Error |
| --- | --- | --- | --- | --- | --- |
| Asian Indian | NA | -2.722954 | 11.673419 | 0.00108 | 0.005798 |
| Chinese | NA | -20.63832 | 15.922805 | 0.009845 | 0.007909 |
| Filipino | NA | **-19.755592** | 8.137222 | **0.009535** | 0.004042 |
| NHW | NA | **-10.86318** | 1.848814 | **0.005189** | 0.000918 |
| Other Asian | NA | **-46.281004** | 8.136652 | **0.022634** | 0.004042 |

Note: Boldface indicates statistical significance (p<0.05) compared to 0

**Appendix Table 6: Parameter estimates of the selected joinpoint model for shingles** **vaccination**

| Race/Ethnicity | Joinpoint (95% CI) | Intercept Estimate | Intercept Standard Error | Slope Estimate | Slope Standard Error | Slope Change Estimate | Slope Change Standard Error |
| --- | --- | --- | --- | --- | --- | --- | --- |
| Asian Indian | NA | **-291.8756** | 101.368444 | **0.144022** | 0.050308 | NA | NA |
| Chinese | NA | -1080.968646 | 624.452106 | 0.536785 | 0.310811 | NA | NA |
| Chinese | 2011 (2010, 2016) | -76.003576 | 73.288668 | 0.037051 | 0.03637 | -0.499734 | 0.312932 |
| Filipino | NA | **-294.956639** | 72.694754 | **0.145699** | 0.036079 | NA | NA |
| NHW | NA | **-419.152831** | 42.619105 | **0.207573** | 0.02119 | NA | NA |
| NHW | 2014 (2012, 2016) | **-93.798362** | 38.156429 | 0.046026 | 0.018921 | **-0.161546** | 0.028408 |
| Other Asian | NA | **-228.89005** | 45.996108 | **0.11295** | 0.022837 | NA | NA |

Note: Boldface indicates statistical significance (p<0.05) compared to 0

**Appendix Table 7: Estimated AAPC (Average Annual Percent Change) from the joinpoint regression model for HPV vaccination**

| Race/Ethnicity | Segment Start Year | Segment End Year | AAPC (95% CI) |
| --- | --- | --- | --- |
| Asian Indian | 2008 | 2018 | **10.1** (2.7, 18.1) |
| Chinese | 2008 | 2018 | **11.7** (6.1, 17.5) |
| Filipino | 2008 | 2018 | **13.5** (8.0, 19.2) |
| NHW | 2008 | 2018 | **11.3** (8.3, 14.3) |
| Other Asian | 2008 | 2018 | **12.5** (7.2, 18.1) |

Note: Boldface indicates statistical significance (p<0.05) compared to AAPC=0

**Appendix Table 8: Estimated APC (Annual Percent Change) from the joinpoint regression model for HPV vaccination**

| Race/Ethnicity | Segment Start (Year) | Segment End (Year) | APC (95% CI) |
| --- | --- | --- | --- |
| Asian Indian | 2008 | 2018 | **10.1** (2.7, 18.1) |
| Chinse | 2008 | 2018 | **11.7** (6.1, 17.5) |
| Filipino | 2008 | 2018 | **13.5** (8, 19.2) |
| NHW | 2008 | 2012 | **20.6** (11.5, 30.4) |
| NHW | 2012 | 2018 | **5.4** (3.3, 7.6) |
| Other Asian | 2008 | 2018 | **12.5** (7.2, 18.1) |

Note: Boldface indicates statistical significance (p<0.05) compared to APC=0

**Appendix Table 9: Estimated AAPC (Average Annual Percent Change) from the joinpoint regression model for hepatitis B vaccination**

| Race/Ethnicity | Start Year | End Year | AAPC (95% CI) |
| --- | --- | --- | --- |
| Asian Indian | 2006 | 2018 | 0.9 (-5.4, 7.6) |
| Chinese | 2006 | 2018 | 1.6 (-1.7, 4.9) |
| Filipino | 2006 | 2018 | 1.0 (-0.7, 2.7) |
| NHW | 2006 | 2018 | **2.2** (0.8, 3.6) |
| Other Asian | 2006 | 2018 | **5.2** (1.9, 8.5) |

Note: Boldface indicates statistical significance (p<0.05) compared to AAPC=0

**Appendix Table 10: Estimated APC (Annual Percent Change) from the joinpoint regression model for hepatitis B vaccination**

| Race/Ethnicity | Segment Start Year | Segment End Year | APC (95% CI) |
| --- | --- | --- | --- |
| Asian Indian | 2006 | 2012 | 4.1 (-1.2, 9.7) |
| Asian Indian | 2012 | 2015 | -9.5 (-31.2, 18.9) |
| Asian Indian | 2015 | 2018 | 5.7 (-10.5, 24.7) |
| Chinese | 2006 | 2012 | **6.7** (0.8, 12.8) |
| Chinese | 2012 | 2018 | -3.3 (-8.1, 1.8) |
| Filipino | 2006 | 2018 | 1.0 (-0.7, 2.7) |
| NHW | 2006 | 2011 | **3.5** (1.4, 5.7) |
| NHW | 2011 | 2016 | -1.9 (-4.3, 0.4) |
| NHW | 2016 | 2018 | **9.7** (1.6, 18.5) |
| Other Asian | 2006 | 2009 | 11.3 (-0.6, 24.6) |
| Other Asian | 2009 | 2016 | -1.0 (-3.5, 1.6) |
| Other Asian | 2016 | 2018 | **19.1** (1.5, 39.6) |

Note: Boldface indicates statistical significance (p<0.05) compared to APC=0

**Appendix Table 11: Estimated AAPC (Average Annual Percent Change) from the joinpoint regression model for pneumococcal vaccination**

| Race/Ethnicity | Start Year | End Year | AAPC (95% CI) |
| --- | --- | --- | --- |
| Asian Indian | 2006 | 2018 | 1.5 (-2.0, 5.2) |
| Chinese | 2006 | 2018 | 3.2 (-0.4, 6.9) |
| Filipino | 2006 | 2018 | **4.4** (2.5, 6.3) |
| NHW | 2006 | 2018 | **1.3** (0.9, 1.8) |
| Other Asian | 2006 | 2018 | 1.4 (-1.6, 4.5) |

Note: Boldface indicates statistical significance (p<0.05) compared to AAPC=0

**Appendix Table 12: Estimated APC (Annual Percent Change) from the joinpoint regression model for pneumococcal vaccination**

| Race/Ethnicity | Segment Start Year | Segment End Year | APC (95% CI) |
| --- | --- | --- | --- |
| Asian Indian | 2006 | 2018 | 1.5 (-2.0, 5.2) |
| Chinese | 2006 | 2018 | 3.2 (-0.4, 6.9) |
| Filipino | 2006 | 2018 | **4.4** (2.5, 6.3) |
| NHW | 2006 | 2018 | **1.3** (0.9, 1.8) |
| Other Asian | 2006 | 2018 | 1.4 (-1.6, 4.5) |

Note: Boldface indicates statistical significance (p<0.05) compared to APC=0

**Appendix Table 13: Estimated AAPC (Average Annual Percent Change) from the joinpoint regression model for influenza vaccination**

| Race/Ethnicity | Start Year | End Year | AAPC (95% CI) |
| --- | --- | --- | --- |
| Asian Indian | 2006 | 2018 | **9.6** (4.6, 14.8) |
| Chinese | 2006 | 2018 | **4.2** (1.8, 6.6) |
| Filipino | 2006 | 2018 | **4.0** (2.1, 5.9) |
| NHW | 2006 | 2018 | **3.0** (2.1, 4.0) |
| Other Asian | 2006 | 2018 | **4.2** (2.9, 5.6) |

Note: Boldface indicates statistical significance (p<0.05) compared to AAPC=0

**Appendix Table 14: Estimated APC (Annual Percent Change) from the joinpoint regression model for influenza vaccination**

| Race/Ethnicity | Segment Start Year | Segment End Year | APC (95% CI) |
| --- | --- | --- | --- |
| Asian Indian | 2006 | 2009 | **25.1** (1.3, 54.5) |
| Asian Indian | 2009 | 2018 | **4.9** (2.8, 6.9) |
| Chinese | 2006 | 2010 | **10.1** (2.5, 18.4) |
| Chinese | 2010 | 2018 | 1.3 (-0.5, 3.1) |
| Filipino | 2006 | 2018 | **4.0** (2.1, 5.9) |
| NHW | 2006 | 2014 | **4.2** (3.1, 5.4) |
| NHW | 2014 | 2018 | 0.6 (-1.8, 3.1) |
| Other Asian | 2006 | 2014 | **6.1** (4.6, 7.7) |
| Other Asian | 2014 | 2018 | 0.5 (-2.9, 4.0) |

Note: Boldface indicates statistical significance (p<0.05) compared to APC=0

**Appendix Table 15: Estimated AAPC (Average Annual Percent Change) from the joinpoint regression model for tetanus diphtheria** **vaccination**

| Race/Ethnicity | Start Year | End Year | AAPC (95% CI) |
| --- | --- | --- | --- |
| Asian Indian | 2008 | 2018 | 0.1 (-1.2, 1.4) |
| Chinse | 2008 | 2018 | 1.0 (-0.8, 2.8) |
| Filipino | 2008 | 2018 | **1.0** (0, 1.9) |
| NHW | 2008 | 2018 | **0.5** (0.3, 0.7) |
| Other Asian | 2008 | 2018 | **2.3** (1.4, 3.2) |

Note: Boldface indicates statistical significance (p<0.05) compared to AAPC=0

**Appendix Table 16: Estimated APC (Annual Percent Change) from the joinpoint regression model for tetanus diphtheria** **vaccination**

| Race/Ethnicity | Start Year | End Year | APC (95% CI) |
| --- | --- | --- | --- |
| Asian Indian | 2008 | 2018 | 0.1 (-1.2, 1.4) |
| Chinese | 2008 | 2018 | 1.0 (-0.8, 2.8) |
| Filipino | 2008 | 2018 | **1.0** (0, 1.9) |
| NHW | 2008 | 2018 | **0.5** (0.3, 0.7) |
| Other Asian | 2008 | 2018 | **2.3** (1.4, 3.2) |

Note: Boldface indicates statistical significance (p<0.05) compared to APC=0

**Appendix Table 17: Estimated AAPC (Average Annual Percent Change) from the joinpoint regression model for shingles** **vaccination**

| Race/Ethnicity | Start Year | End Year | AAPC (95% CI) |
| --- | --- | --- | --- |
| Asian Indian | 2008 | 2018 | **15.5** (2.8, 29.7) |
| Chinese | 2008 | 2018 | 20.6 (-0.2, 45.7) |
| Filipino | 2008 | 2018 | **15.7** (6.6, 25.5) |
| NHW | 2008 | 2018 | **15.4** (12.1, 18.8) |
| Other Asian | 2008 | 2018 | **12.0** (6.3, 17.9) |

Note: Boldface indicates statistical significance (p<0.05) compared to AAPC=0

**Appendix Table 18: Estimated APC (Annual Percent Change) from the joinpoint regression model for shingles** **vaccination**

| Race/Ethnicity | Segment Start Year | Segment End Year | APC (95% CI) |
| --- | --- | --- | --- |
| Asian Indian | 2008 | 2018 | **15.5** (2.8, 29.7) |
| Chinese | 2008 | 2011 | 71 (-20.0, 265.9) |
| Chinese | 2011 | 2018 | 3.8 (-5.1, 13.4) |
| Filipino | 2008 | 2018 | **15.7** (6.6, 25.5) |
| NHW | 2008 | 2014 | **23.1** (16.9, 29.6) |
| NHW | 2014 | 2018 | 4.7 (0, 9.7) |
| Other Asian | 2008 | 2018 | **12.0 (**6.3, 17.9) |

Note: Boldface indicates statistical significance (p<0.05) compared to APC=0

**Appendix Table 19**: **Raw data on all vaccines and racial/ethnic groups for fitting Joinpoint regressions**

| Race/Ethnicity | Year | Vaccination Rate | Standard Error | Vaccine |
| --- | --- | --- | --- | --- |
| Asian Indian | 2008 | 0.02 | 0.02 | Shingles Vaccine |
| Asian Indian | 2010 | 0.11 | 0.05 | Shingles Vaccine |
| Asian Indian | 2011 | 0.02 | 0.02 | Shingles Vaccine |
| Asian Indian | 2012 | 0.18 | 0.06 | Shingles Vaccine |
| Asian Indian | 2013 | 0.12 | 0.05 | Shingles Vaccine |
| Asian Indian | 2014 | 0.23 | 0.07 | Shingles Vaccine |
| Asian Indian | 2015 | 0.1 | 0.05 | Shingles Vaccine |
| Asian Indian | 2016 | 0.15 | 0.07 | Shingles Vaccine |
| Asian Indian | 2017 | 0.18 | 0.07 | Shingles Vaccine |
| Asian Indian | 2018 | 0.33 | 0.07 | Shingles Vaccine |
| Chinese | 2008 | 0.07 | 0.04 | Shingles Vaccine |
| Chinese | 2009 | 0.05 | 0.03 | Shingles Vaccine |
| Chinese | 2010 | 0.08 | 0.04 | Shingles Vaccine |
| Chinese | 2011 | 0.24 | 0.05 | Shingles Vaccine |
| Chinese | 2012 | 0.21 | 0.05 | Shingles Vaccine |
| Chinese | 2013 | 0.26 | 0.05 | Shingles Vaccine |
| Chinese | 2014 | 0.17 | 0.05 | Shingles Vaccine |
| Chinese | 2015 | 0.34 | 0.06 | Shingles Vaccine |
| Chinese | 2016 | 0.29 | 0.08 | Shingles Vaccine |
| Chinese | 2017 | 0.27 | 0.05 | Shingles Vaccine |
| Chinese | 2018 | 0.26 | 0.06 | Shingles Vaccine |
| Filipino | 2008 | 0.01 | 0.01 | Shingles Vaccine |
| Filipino | 2009 | 0.1 | 0.03 | Shingles Vaccine |
| Filipino | 2010 | 0.08 | 0.03 | Shingles Vaccine |
| Filipino | 2011 | 0.08 | 0.03 | Shingles Vaccine |
| Filipino | 2012 | 0.21 | 0.04 | Shingles Vaccine |
| Filipino | 2013 | 0.24 | 0.05 | Shingles Vaccine |
| Filipino | 2014 | 0.2 | 0.04 | Shingles Vaccine |
| Filipino | 2015 | 0.28 | 0.04 | Shingles Vaccine |
| Filipino | 2016 | 0.3 | 0.05 | Shingles Vaccine |
| Filipino | 2017 | 0.38 | 0.05 | Shingles Vaccine |
| Filipino | 2018 | 0.26 | 0.05 | Shingles Vaccine |
| NHW | 2008 | 0.08 | 0 | Shingles Vaccine |
| NHW | 2009 | 0.11 | 0.01 | Shingles Vaccine |
| NHW | 2010 | 0.16 | 0.01 | Shingles Vaccine |
| NHW | 2011 | 0.18 | 0.01 | Shingles Vaccine |
| NHW | 2012 | 0.23 | 0.01 | Shingles Vaccine |
| NHW | 2013 | 0.27 | 0.01 | Shingles Vaccine |
| NHW | 2014 | 0.32 | 0.01 | Shingles Vaccine |
| NHW | 2015 | 0.34 | 0.01 | Shingles Vaccine |
| NHW | 2016 | 0.37 | 0.01 | Shingles Vaccine |
| NHW | 2017 | 0.39 | 0.01 | Shingles Vaccine |
| NHW | 2018 | 0.39 | 0.01 | Shingles Vaccine |
| Asian Indian | 2008 | 0.05 | 0.03 | Shingles Vaccine |
| Asian Indian | 2009 | 0.09 | 0.02 | Shingles Vaccine |
| Asian Indian | 2010 | 0.18 | 0.03 | Shingles Vaccine |
| Asian Indian | 2011 | 0.19 | 0.03 | Shingles Vaccine |
| Asian Indian | 2012 | 0.19 | 0.03 | Shingles Vaccine |
| Asian Indian | 2013 | 0.24 | 0.04 | Shingles Vaccine |
| Asian Indian | 2014 | 0.21 | 0.04 | Shingles Vaccine |
| Asian Indian | 2015 | 0.32 | 0.04 | Shingles Vaccine |
| Asian Indian | 2016 | 0.23 | 0.04 | Shingles Vaccine |
| Asian Indian | 2017 | 0.4 | 0.05 | Shingles Vaccine |
| Asian Indian | 2018 | 0.31 | 0.05 | Shingles Vaccine |
| Asian Indian | 2006 | 0.17 | 0.03 | Influenza Vaccine |
| Asian Indian | 2007 | 0.23 | 0.03 | Influenza Vaccine |
| Asian Indian | 2008 | 0.24 | 0.03 | Influenza Vaccine |
| Asian Indian | 2009 | 0.34 | 0.03 | Influenza Vaccine |
| Asian Indian | 2010 | 0.32 | 0.03 | Influenza Vaccine |
| Asian Indian | 2011 | 0.38 | 0.03 | Influenza Vaccine |
| Asian Indian | 2012 | 0.37 | 0.03 | Influenza Vaccine |
| Asian Indian | 2013 | 0.42 | 0.03 | Influenza Vaccine |
| Asian Indian | 2014 | 0.43 | 0.03 | Influenza Vaccine |
| Asian Indian | 2015 | 0.48 | 0.02 | Influenza Vaccine |
| Asian Indian | 2016 | 0.43 | 0.03 | Influenza Vaccine |
| Asian Indian | 2017 | 0.46 | 0.03 | Influenza Vaccine |
| Asian Indian | 2018 | 0.5 | 0.03 | Influenza Vaccine |
| Chinese | 2006 | 0.29 | 0.03 | Influenza Vaccine |
| Chinese | 2007 | 0.29 | 0.04 | Influenza Vaccine |
| Chinese | 2008 | 0.29 | 0.03 | Influenza Vaccine |
| Chinese | 2009 | 0.37 | 0.03 | Influenza Vaccine |
| Chinese | 2010 | 0.41 | 0.03 | Influenza Vaccine |
| Chinese | 2011 | 0.4 | 0.03 | Influenza Vaccine |
| Chinese | 2012 | 0.41 | 0.03 | Influenza Vaccine |
| Chinese | 2013 | 0.43 | 0.03 | Influenza Vaccine |
| Chinese | 2014 | 0.44 | 0.03 | Influenza Vaccine |
| Chinese | 2015 | 0.4 | 0.03 | Influenza Vaccine |
| Chinese | 2016 | 0.45 | 0.04 | Influenza Vaccine |
| Chinese | 2017 | 0.43 | 0.04 | Influenza Vaccine |
| Chinese | 2018 | 0.43 | 0.03 | Influenza Vaccine |
| Filipino | 2006 | 0.31 | 0.03 | Influenza Vaccine |
| Filipino | 2007 | 0.31 | 0.03 | Influenza Vaccine |
| Filipino | 2008 | 0.36 | 0.04 | Influenza Vaccine |
| Filipino | 2009 | 0.48 | 0.03 | Influenza Vaccine |
| Filipino | 2010 | 0.46 | 0.03 | Influenza Vaccine |
| Filipino | 2011 | 0.43 | 0.03 | Influenza Vaccine |
| Filipino | 2012 | 0.47 | 0.03 | Influenza Vaccine |
| Filipino | 2013 | 0.48 | 0.02 | Influenza Vaccine |
| Filipino | 2014 | 0.52 | 0.03 | Influenza Vaccine |
| Filipino | 2015 | 0.57 | 0.03 | Influenza Vaccine |
| Filipino | 2016 | 0.48 | 0.03 | Influenza Vaccine |
| Filipino | 2017 | 0.52 | 0.04 | Influenza Vaccine |
| Filipino | 2018 | 0.55 | 0.04 | Influenza Vaccine |
| NHW | 2006 | 0.33 | 0.01 | Influenza Vaccine |
| NHW | 2007 | 0.35 | 0.01 | Influenza Vaccine |
| NHW | 2008 | 0.39 | 0.01 | Influenza Vaccine |
| NHW | 2009 | 0.4 | 0 | Influenza Vaccine |
| NHW | 2010 | 0.4 | 0.01 | Influenza Vaccine |
| NHW | 2011 | 0.42 | 0 | Influenza Vaccine |
| NHW | 2012 | 0.42 | 0 | Influenza Vaccine |
| NHW | 2013 | 0.46 | 0 | Influenza Vaccine |
| NHW | 2014 | 0.48 | 0 | Influenza Vaccine |
| NHW | 2015 | 0.49 | 0 | Influenza Vaccine |
| NHW | 2016 | 0.47 | 0 | Influenza Vaccine |
| NHW | 2017 | 0.48 | 0 | Influenza Vaccine |
| NHW | 2018 | 0.5 | 0 | Influenza Vaccine |
| Other Asian | 2006 | 0.31 | 0.02 | Influenza Vaccine |
| Other Asian | 2007 | 0.3 | 0.03 | Influenza Vaccine |
| Other Asian | 2008 | 0.36 | 0.03 | Influenza Vaccine |
| Other Asian | 2009 | 0.33 | 0.02 | Influenza Vaccine |
| Other Asian | 2010 | 0.39 | 0.02 | Influenza Vaccine |
| Other Asian | 2011 | 0.41 | 0.02 | Influenza Vaccine |
| Other Asian | 2012 | 0.42 | 0.02 | Influenza Vaccine |
| Other Asian | 2013 | 0.44 | 0.02 | Influenza Vaccine |
| Other Asian | 2014 | 0.5 | 0.02 | Influenza Vaccine |
| Other Asian | 2015 | 0.49 | 0.02 | Influenza Vaccine |
| Other Asian | 2016 | 0.48 | 0.03 | Influenza Vaccine |
| Other Asian | 2017 | 0.5 | 0.03 | Influenza Vaccine |
| Other Asian | 2018 | 0.48 | 0.03 | Influenza Vaccine |
| Asian Indian | 2006 | 0.45 | 0.04 | Hepatitis B Vaccine |
| Asian Indian | 2007 | 0.37 | 0.04 | Hepatitis B Vaccine |
| Asian Indian | 2008 | 0.44 | 0.04 | Hepatitis B Vaccine |
| Asian Indian | 2009 | 0.42 | 0.04 | Hepatitis B Vaccine |
| Asian Indian | 2010 | 0.44 | 0.04 | Hepatitis B Vaccine |
| Asian Indian | 2011 | 0.52 | 0.03 | Hepatitis B Vaccine |
| Asian Indian | 2012 | 0.51 | 0.03 | Hepatitis B Vaccine |
| Asian Indian | 2013 | 0.44 | 0.03 | Hepatitis B Vaccine |
| Asian Indian | 2014 | 0.43 | 0.03 | Hepatitis B Vaccine |
| Asian Indian | 2015 | 0.38 | 0.03 | Hepatitis B Vaccine |
| Asian Indian | 2016 | 0.4 | 0.03 | Hepatitis B Vaccine |
| Asian Indian | 2017 | 0.39 | 0.03 | Hepatitis B Vaccine |
| Asian Indian | 2018 | 0.47 | 0.04 | Hepatitis B Vaccine |
| Chinese | 2006 | 0.32 | 0.03 | Hepatitis B Vaccine |
| Chinese | 2007 | 0.37 | 0.04 | Hepatitis B Vaccine |
| Chinese | 2008 | 0.32 | 0.04 | Hepatitis B Vaccine |
| Chinese | 2009 | 0.31 | 0.03 | Hepatitis B Vaccine |
| Chinese | 2010 | 0.37 | 0.04 | Hepatitis B Vaccine |
| Chinese | 2011 | 0.47 | 0.03 | Hepatitis B Vaccine |
| Chinese | 2012 | 0.44 | 0.03 | Hepatitis B Vaccine |
| Chinese | 2013 | 0.45 | 0.03 | Hepatitis B Vaccine |
| Chinese | 2014 | 0.41 | 0.03 | Hepatitis B Vaccine |
| Chinese | 2015 | 0.43 | 0.03 | Hepatitis B Vaccine |
| Chinese | 2016 | 0.34 | 0.03 | Hepatitis B Vaccine |
| Chinese | 2017 | 0.4 | 0.03 | Hepatitis B Vaccine |
| Chinese | 2018 | 0.37 | 0.03 | Hepatitis B Vaccine |
| Filipino | 2006 | 0.37 | 0.04 | Hepatitis B Vaccine |
| Filipino | 2007 | 0.35 | 0.04 | Hepatitis B Vaccine |
| Filipino | 2008 | 0.44 | 0.04 | Hepatitis B Vaccine |
| Filipino | 2009 | 0.33 | 0.03 | Hepatitis B Vaccine |
| Filipino | 2010 | 0.42 | 0.03 | Hepatitis B Vaccine |
| Filipino | 2011 | 0.45 | 0.03 | Hepatitis B Vaccine |
| Filipino | 2012 | 0.39 | 0.03 | Hepatitis B Vaccine |
| Filipino | 2013 | 0.37 | 0.03 | Hepatitis B Vaccine |
| Filipino | 2014 | 0.4 | 0.03 | Hepatitis B Vaccine |
| Filipino | 2015 | 0.44 | 0.03 | Hepatitis B Vaccine |
| Filipino | 2016 | 0.4 | 0.03 | Hepatitis B Vaccine |
| Filipino | 2017 | 0.42 | 0.04 | Hepatitis B Vaccine |
| Filipino | 2018 | 0.44 | 0.04 | Hepatitis B Vaccine |
| NHW | 2006 | 0.26 | 0.01 | Hepatitis B Vaccine |
| NHW | 2007 | 0.27 | 0.01 | Hepatitis B Vaccine |
| NHW | 2008 | 0.29 | 0.01 | Hepatitis B Vaccine |
| NHW | 2009 | 0.29 | 0 | Hepatitis B Vaccine |
| NHW | 2010 | 0.3 | 0 | Hepatitis B Vaccine |
| NHW | 2011 | 0.31 | 0 | Hepatitis B Vaccine |
| NHW | 2012 | 0.31 | 0 | Hepatitis B Vaccine |
| NHW | 2013 | 0.29 | 0 | Hepatitis B Vaccine |
| NHW | 2014 | 0.29 | 0 | Hepatitis B Vaccine |
| NHW | 2015 | 0.29 | 0 | Hepatitis B Vaccine |
| NHW | 2016 | 0.29 | 0 | Hepatitis B Vaccine |
| NHW | 2017 | 0.3 | 0 | Hepatitis B Vaccine |
| NHW | 2018 | 0.35 | 0.01 | Hepatitis B Vaccine |
| Other Asian | 2006 | 0.27 | 0.02 | Hepatitis B Vaccine |
| Other Asian | 2007 | 0.31 | 0.02 | Hepatitis B Vaccine |
| Other Asian | 2008 | 0.31 | 0.03 | Hepatitis B Vaccine |
| Other Asian | 2009 | 0.38 | 0.03 | Hepatitis B Vaccine |
| Other Asian | 2010 | 0.37 | 0.02 | Hepatitis B Vaccine |
| Other Asian | 2011 | 0.36 | 0.02 | Hepatitis B Vaccine |
| Other Asian | 2012 | 0.38 | 0.02 | Hepatitis B Vaccine |
| Other Asian | 2013 | 0.33 | 0.02 | Hepatitis B Vaccine |
| Other Asian | 2014 | 0.35 | 0.02 | Hepatitis B Vaccine |
| Other Asian | 2015 | 0.35 | 0.02 | Hepatitis B Vaccine |
| Other Asian | 2016 | 0.35 | 0.03 | Hepatitis B Vaccine |
| Other Asian | 2017 | 0.42 | 0.03 | Hepatitis B Vaccine |
| Other Asian | 2018 | 0.49 | 0.03 | Hepatitis B Vaccine |
| Asian Indian | 2008 | 0.03 | 0.03 | HPV Vaccine |
| Asian Indian | 2009 | 0.07 | 0.04 | HPV Vaccine |
| Asian Indian | 2010 | 0.08 | 0.05 | HPV Vaccine |
| Asian Indian | 2011 | 0.1 | 0.04 | HPV Vaccine |
| Asian Indian | 2012 | 0.07 | 0.03 | HPV Vaccine |
| Asian Indian | 2013 | 0.08 | 0.05 | HPV Vaccine |
| Asian Indian | 2014 | 0.07 | 0.03 | HPV Vaccine |
| Asian Indian | 2015 | 0.11 | 0.05 | HPV Vaccine |
| Asian Indian | 2016 | 0.12 | 0.04 | HPV Vaccine |
| Asian Indian | 2017 | 0.08 | 0.03 | HPV Vaccine |
| Asian Indian | 2018 | 0.19 | 0.05 | HPV Vaccine |
| Chinese | 2008 | 0.07 | 0.05 | HPV Vaccine |
| Chinese | 2009 | 0.2 | 0.09 | HPV Vaccine |
| Chinese | 2010 | 0.14 | 0.06 | HPV Vaccine |
| Chinese | 2011 | 0.2 | 0.06 | HPV Vaccine |
| Chinese | 2012 | 0.22 | 0.05 | HPV Vaccine |
| Chinese | 2013 | 0.36 | 0.07 | HPV Vaccine |
| Chinese | 2014 | 0.22 | 0.05 | HPV Vaccine |
| Chinese | 2015 | 0.33 | 0.06 | HPV Vaccine |
| Chinese | 2016 | 0.37 | 0.07 | HPV Vaccine |
| Chinese | 2017 | 0.37 | 0.07 | HPV Vaccine |
| Chinese | 2018 | 0.45 | 0.07 | HPV Vaccine |
| Filipino | 2008 | 0.11 | 0.1 | HPV Vaccine |
| Filipino | 2009 | 0.14 | 0.09 | HPV Vaccine |
| Filipino | 2010 | 0.1 | 0.05 | HPV Vaccine |
| Filipino | 2011 | 0.27 | 0.07 | HPV Vaccine |
| Filipino | 2012 | 0.2 | 0.07 | HPV Vaccine |
| Filipino | 2013 | 0.27 | 0.08 | HPV Vaccine |
| Filipino | 2014 | 0.23 | 0.06 | HPV Vaccine |
| Filipino | 2015 | 0.34 | 0.09 | HPV Vaccine |
| Filipino | 2016 | 0.31 | 0.08 | HPV Vaccine |
| Filipino | 2017 | 0.41 | 0.09 | HPV Vaccine |
| Filipino | 2018 | 0.49 | 0.1 | HPV Vaccine |
| NHW | 2008 | 0.13 | 0.01 | HPV Vaccine |
| NHW | 2009 | 0.18 | 0.02 | HPV Vaccine |
| NHW | 2010 | 0.19 | 0.01 | HPV Vaccine |
| NHW | 2011 | 0.24 | 0.01 | HPV Vaccine |
| NHW | 2012 | 0.28 | 0.01 | HPV Vaccine |
| NHW | 2013 | 0.31 | 0.01 | HPV Vaccine |
| NHW | 2014 | 0.32 | 0.01 | HPV Vaccine |
| NHW | 2015 | 0.32 | 0.01 | HPV Vaccine |
| NHW | 2016 | 0.35 | 0.01 | HPV Vaccine |
| NHW | 2017 | 0.38 | 0.01 | HPV Vaccine |
| NHW | 2018 | 0.39 | 0.01 | HPV Vaccine |
| Other Asian | 2008 | 0.08 | 0.04 | HPV Vaccine |
| Other Asian | 2009 | 0.06 | 0.03 | HPV Vaccine |
| Other Asian | 2010 | 0.16 | 0.05 | HPV Vaccine |
| Other Asian | 2011 | 0.18 | 0.04 | HPV Vaccine |
| Other Asian | 2012 | 0.22 | 0.05 | HPV Vaccine |
| Other Asian | 2013 | 0.17 | 0.04 | HPV Vaccine |
| Other Asian | 2014 | 0.24 | 0.05 | HPV Vaccine |
| Other Asian | 2015 | 0.22 | 0.05 | HPV Vaccine |
| Other Asian | 2016 | 0.27 | 0.05 | HPV Vaccine |
| Other Asian | 2017 | 0.38 | 0.08 | HPV Vaccine |
| Other Asian | 2018 | 0.33 | 0.06 | HPV Vaccine |
| Asian Indian | 2006 | 0.36 | 0.15 | Pneumococcal Vaccine |
| Asian Indian | 2007 | 0.25 | 0.15 | Pneumococcal Vaccine |
| Asian Indian | 2008 | 0.57 | 0.11 | Pneumococcal Vaccine |
| Asian Indian | 2009 | 0.42 | 0.14 | Pneumococcal Vaccine |
| Asian Indian | 2010 | 0.7 | 0.1 | Pneumococcal Vaccine |
| Asian Indian | 2011 | 0.36 | 0.1 | Pneumococcal Vaccine |
| Asian Indian | 2012 | 0.35 | 0.1 | Pneumococcal Vaccine |
| Asian Indian | 2013 | 0.53 | 0.11 | Pneumococcal Vaccine |
| Asian Indian | 2014 | 0.53 | 0.09 | Pneumococcal Vaccine |
| Asian Indian | 2015 | 0.5 | 0.08 | Pneumococcal Vaccine |
| Asian Indian | 2016 | 0.54 | 0.11 | Pneumococcal Vaccine |
| Asian Indian | 2017 | 0.61 | 0.12 | Pneumococcal Vaccine |
| Asian Indian | 2018 | 0.62 | 0.08 | Pneumococcal Vaccine |
| Chinese | 2006 | 0.26 | 0.08 | Pneumococcal Vaccine |
| Chinese | 2007 | 0.45 | 0.09 | Pneumococcal Vaccine |
| Chinese | 2008 | 0.37 | 0.07 | Pneumococcal Vaccine |
| Chinese | 2009 | 0.31 | 0.08 | Pneumococcal Vaccine |
| Chinese | 2010 | 0.3 | 0.06 | Pneumococcal Vaccine |
| Chinese | 2011 | 0.47 | 0.07 | Pneumococcal Vaccine |
| Chinese | 2012 | 0.39 | 0.07 | Pneumococcal Vaccine |
| Chinese | 2013 | 0.38 | 0.06 | Pneumococcal Vaccine |
| Chinese | 2014 | 0.34 | 0.06 | Pneumococcal Vaccine |
| Chinese | 2015 | 0.6 | 0.07 | Pneumococcal Vaccine |
| Chinese | 2016 | 0.45 | 0.08 | Pneumococcal Vaccine |
| Chinese | 2017 | 0.47 | 0.07 | Pneumococcal Vaccine |
| Chinese | 2018 | 0.45 | 0.08 | Pneumococcal Vaccine |
| Filipino | 2006 | 0.27 | 0.08 | Pneumococcal Vaccine |
| Filipino | 2007 | 0.41 | 0.09 | Pneumococcal Vaccine |
| Filipino | 2008 | 0.44 | 0.09 | Pneumococcal Vaccine |
| Filipino | 2009 | 0.48 | 0.07 | Pneumococcal Vaccine |
| Filipino | 2010 | 0.57 | 0.06 | Pneumococcal Vaccine |
| Filipino | 2011 | 0.49 | 0.06 | Pneumococcal Vaccine |
| Filipino | 2012 | 0.47 | 0.05 | Pneumococcal Vaccine |
| Filipino | 2013 | 0.56 | 0.06 | Pneumococcal Vaccine |
| Filipino | 2014 | 0.5 | 0.06 | Pneumococcal Vaccine |
| Filipino | 2015 | 0.52 | 0.07 | Pneumococcal Vaccine |
| Filipino | 2016 | 0.63 | 0.05 | Pneumococcal Vaccine |
| Filipino | 2017 | 0.66 | 0.06 | Pneumococcal Vaccine |
| Filipino | 2018 | 0.68 | 0.05 | Pneumococcal Vaccine |
| NHW | 2006 | 0.62 | 0.01 | Pneumococcal Vaccine |
| NHW | 2007 | 0.63 | 0.01 | Pneumococcal Vaccine |
| NHW | 2008 | 0.64 | 0.01 | Pneumococcal Vaccine |
| NHW | 2009 | 0.65 | 0.01 | Pneumococcal Vaccine |
| NHW | 2010 | 0.63 | 0.01 | Pneumococcal Vaccine |
| NHW | 2011 | 0.66 | 0.01 | Pneumococcal Vaccine |
| NHW | 2012 | 0.65 | 0.01 | Pneumococcal Vaccine |
| NHW | 2013 | 0.64 | 0.01 | Pneumococcal Vaccine |
| NHW | 2014 | 0.66 | 0.01 | Pneumococcal Vaccine |
| NHW | 2015 | 0.68 | 0.01 | Pneumococcal Vaccine |
| NHW | 2016 | 0.71 | 0.01 | Pneumococcal Vaccine |
| NHW | 2017 | 0.73 | 0.01 | Pneumococcal Vaccine |
| NHW | 2018 | 0.72 | 0.01 | Pneumococcal Vaccine |
| Other Asian | 2006 | 0.47 | 0.05 | Pneumococcal Vaccine |
| Other Asian | 2007 | 0.25 | 0.06 | Pneumococcal Vaccine |
| Other Asian | 2008 | 0.47 | 0.06 | Pneumococcal Vaccine |
| Other Asian | 2009 | 0.48 | 0.06 | Pneumococcal Vaccine |
| Other Asian | 2010 | 0.47 | 0.05 | Pneumococcal Vaccine |
| Other Asian | 2011 | 0.34 | 0.04 | Pneumococcal Vaccine |
| Other Asian | 2012 | 0.41 | 0.06 | Pneumococcal Vaccine |
| Other Asian | 2013 | 0.36 | 0.04 | Pneumococcal Vaccine |
| Other Asian | 2014 | 0.49 | 0.05 | Pneumococcal Vaccine |
| Other Asian | 2015 | 0.39 | 0.05 | Pneumococcal Vaccine |
| Other Asian | 2016 | 0.46 | 0.06 | Pneumococcal Vaccine |
| Other Asian | 2017 | 0.6 | 0.06 | Pneumococcal Vaccine |
| Other Asian | 2018 | 0.47 | 0.05 | Pneumococcal Vaccine |
| Asian Indian | 2008 | 0.56 | 0.04 | Tetanus Vaccine |
| Asian Indian | 2009 | 0.55 | 0.04 | Tetanus Vaccine |
| Asian Indian | 2010 | 0.53 | 0.04 | Tetanus Vaccine |
| Asian Indian | 2011 | 0.56 | 0.03 | Tetanus Vaccine |
| Asian Indian | 2012 | 0.63 | 0.03 | Tetanus Vaccine |
| Asian Indian | 2013 | 0.57 | 0.03 | Tetanus Vaccine |
| Asian Indian | 2014 | 0.62 | 0.03 | Tetanus Vaccine |
| Asian Indian | 2015 | 0.59 | 0.03 | Tetanus Vaccine |
| Asian Indian | 2016 | 0.57 | 0.03 | Tetanus Vaccine |
| Asian Indian | 2017 | 0.55 | 0.03 | Tetanus Vaccine |
| Asian Indian | 2018 | 0.56 | 0.03 | Tetanus Vaccine |
| Chinese | 2008 | 0.46 | 0.04 | Tetanus Vaccine |
| Chinese | 2009 | 0.37 | 0.04 | Tetanus Vaccine |
| Chinese | 2010 | 0.42 | 0.03 | Tetanus Vaccine |
| Chinese | 2011 | 0.46 | 0.03 | Tetanus Vaccine |
| Chinese | 2012 | 0.41 | 0.02 | Tetanus Vaccine |
| Chinese | 2013 | 0.48 | 0.03 | Tetanus Vaccine |
| Chinese | 2014 | 0.41 | 0.03 | Tetanus Vaccine |
| Chinese | 2015 | 0.42 | 0.03 | Tetanus Vaccine |
| Chinese | 2016 | 0.44 | 0.04 | Tetanus Vaccine |
| Chinese | 2017 | 0.49 | 0.03 | Tetanus Vaccine |
| Chinese | 2018 | 0.47 | 0.04 | Tetanus Vaccine |
| Filipino | 2008 | 0.54 | 0.04 | Tetanus Vaccine |
| Filipino | 2009 | 0.53 | 0.03 | Tetanus Vaccine |
| Filipino | 2010 | 0.56 | 0.03 | Tetanus Vaccine |
| Filipino | 2011 | 0.53 | 0.03 | Tetanus Vaccine |
| Filipino | 2012 | 0.6 | 0.03 | Tetanus Vaccine |
| Filipino | 2013 | 0.58 | 0.03 | Tetanus Vaccine |
| Filipino | 2014 | 0.56 | 0.03 | Tetanus Vaccine |
| Filipino | 2015 | 0.6 | 0.03 | Tetanus Vaccine |
| Filipino | 2016 | 0.57 | 0.03 | Tetanus Vaccine |
| Filipino | 2017 | 0.61 | 0.04 | Tetanus Vaccine |
| Filipino | 2018 | 0.58 | 0.03 | Tetanus Vaccine |
| NHW | 2008 | 0.64 | 0.01 | Tetanus Vaccine |
| NHW | 2009 | 0.64 | 0.01 | Tetanus Vaccine |
| NHW | 2010 | 0.65 | 0.01 | Tetanus Vaccine |
| NHW | 2011 | 0.66 | 0 | Tetanus Vaccine |
| NHW | 2012 | 0.65 | 0 | Tetanus Vaccine |
| NHW | 2013 | 0.66 | 0 | Tetanus Vaccine |
| NHW | 2014 | 0.66 | 0 | Tetanus Vaccine |
| NHW | 2015 | 0.66 | 0.01 | Tetanus Vaccine |
| NHW | 2016 | 0.67 | 0.01 | Tetanus Vaccine |
| NHW | 2017 | 0.68 | 0.01 | Tetanus Vaccine |
| NHW | 2018 | 0.67 | 0.01 | Tetanus Vaccine |
| Other Asian | 2008 | 0.45 | 0.03 | Tetanus Vaccine |
| Other Asian | 2009 | 0.44 | 0.03 | Tetanus Vaccine |
| Other Asian | 2010 | 0.46 | 0.02 | Tetanus Vaccine |
| Other Asian | 2011 | 0.48 | 0.02 | Tetanus Vaccine |
| Other Asian | 2012 | 0.48 | 0.02 | Tetanus Vaccine |
| Other Asian | 2013 | 0.46 | 0.02 | Tetanus Vaccine |
| Other Asian | 2014 | 0.47 | 0.02 | Tetanus Vaccine |
| Other Asian | 2015 | 0.51 | 0.02 | Tetanus Vaccine |
| Other Asian | 2016 | 0.52 | 0.03 | Tetanus Vaccine |
| Other Asian | 2017 | 0.56 | 0.03 | Tetanus Vaccine |
| Other Asian | 2018 | 0.56 | 0.03 | Tetanus Vaccine |

**Appendix Table 20: Multivariable logistic regression on NHW and stratified US-born and Foreign-born Asian subgroups, NHIS 2015-2018**

|  | **HPV vaccine, AOR^a^ (95% CI)** | | **Hepatitis B vaccine, AOR (95% CI)** | | **Pneumococcal vaccine, AOR (95% CI)** | | **Influenza vaccine, AOR (95% CI)** | | **Tetanus Diphtheria vaccine, AOR (95% CI)** | | **Shingles vaccine, AOR (95% CI)** | |
| --- | --- | --- | --- | --- | --- | --- | --- | --- | --- | --- | --- | --- |
|  | **Foreign born Asians** | **US born Asians** | **Foreign born Asians** | **US born Asians** | **Foreign born Asians** | **US born Asians** | **Foreign born Asians** | **US born Asians** | **Foreign born Asians** | **US born Asians** | **Foreign born Asians** | **US born Asians** |
| **Race** |  |  |  |  |  |  |  |  |  |  |  |  |
| NHW | ref | ref | ref | ref | ref | ref | ref | ref | ref | ref | ref | ref |
| All Asian | **0.47 (0.36, 0.60)** | **1.14 (1.84, 1.55)** | **1.18 (1.07, 1.31)** | **1.66 (1.44, 1.90)** | **0.46 (0.35, 0.61)** | 0.73 (0.49, 1.10) | **1.15 (1.05, 1.24)** | **1.69 (1.48, 1.94)** | **0.47 (0.44, 0.52)** | 1.03 (0.88, 1.21) | **0.55 (0.44, 0.69)** | **1.44 (1.01, 2.05)** |
| Chinese | **0.54 (0.36, 0.82)** | 1.30 (0.66-2.56) | 0.97 (0.80-1.16) | **1.65 (1.22-2.23)** | **0.30 (0.18-0.51)** | 1.01 (0.36-3.39) | **0.83 (0.71-0.98)** | **1.81 (1.34-2.45)** | **0.31 (0.26-0.37)** | 1.24 (0.87-1.77) | **0.51 (0.32-0.83)** | 1.00 (0.38-2.65) |
| Filipino | 1.03 (0.48-2.19) | 1.52 (0.78-3.00) | **1.68 (1.37-2.07)** | **1.59 (1.23-2.05)** | 0.67 (0.39-1.15) | **0.54 (0.27-1.10)** | **1.49 (1.18-1.88)** | **1.50 (1.15-1.95)** | **0.61 (0.50-0.75)** | 0.90 (0.68-1.17) | **0.54 (0.34-0.87)** | **1.18 (0.64-2.18)** |
| Asian Indian | **0.212 (0.12-0.40)** | 0.81 (0.39-1.70) | 0.99 (0.84-1.17) | **2.25 (1.41-3.59)** | 0.72 (0.42-1.25) | 6491 (902-46707) * | 1.12 (0.98-1.28) | **2.50 (1.60-3.91)** | **0.54 (0.47-0.63)** | 1.44 (0.86-2.41) | **0.38 (0.22-0.65)** | 3.6e-5 (7.6e-6, 1.7e04) * |
| Other Asian | **0.59 (0.37-0.95)** | 0.99 (0.59-1.65) | **1.41 (1.20-1.66)** | **1.57 (1.26-1.94)** | **0.35 (0.24-0.50)** | 0.83 (0.47-1.46) | **1.32 (1.13-1.54)** | **1.58 (1.29-1.95)** | **0.50 (0.43-0.58)** | 0.96 (0.75-1.24) | 0.73 (0.52-1.04) | **2.05 (1.27-3.31)** |
| **Sex** |  |  |  |  |  |  |  |  |  |  |  |  |
| Female | NA | ref | ref | ref | ref | ref | ref | ref | ref | ref | ref | ref |
| Male | NA | NA | **0.69 (0.67-0.73)** | **0.68 (0.65-0.72)** | **0.83 (0.75-0.92)** | **0.84 (0.76-0.94)** | **0.82 (0.78-0.85)** | **0.81 (0.78-0.85)** | **1.12 (1.17-1.26)** | **1.23 (1.18-1.28)** | **0.75 (0.69-0.80)** | **0.74 (0.69-0.78)** |
| **Age** |  |  |  |  |  |  |  |  |  |  |  |  |
| 19-26 | NA | NA | **ref** | **ref** | NA | NA | **ref** | **ref** | **ref** | **ref** | NA | NA |
| 27-49 | NA | NA | **0.56 (0.52-0.60)** | **0.55 (0.51-0.60)** | NA | NA | 1.05 (0.98-1.14) | 1.05 (0.97-1.13) | **0.72 (0.67-0.78)** | **0.72 (0.67-78)** | NA | NA |
| 50-64 | NA | NA | **0.30 (0.26-0.30)** | **0.28 (0.25-0.30)** | NA | NA | **1.60 (1.48-1.75)** | **1.61 (1.48-1.74)** | **0.68 (0.63-0.73)** | **0.68 (0.63-0.74)** | NA | NA |
| 65+ | NA | NA | **0.13 (0.12-0.14)** | **0.13 (0.11-0.14)** | NA | NA | **4.04 (3.73-4.39)** | **4.07 (3.75 -4.41)** | **0.49 (0.45-0.54)** | **0.49 (0.45-0.54)** | NA | NA |
| Within HPV age-eligible adult females | **0.85 (0.84-0.86)** | **0.85 (0.84-0.86)** | NA | NA | NA | NA | NA | NA | NA | NA | NA | NA |
| Within 60+ (Shingles) | NA | NA | NA | NA | NA | NA | NA | NA | NA | NA | **1.04 (1.03-1.05)** | **1.04 (1.03-1.05)** |
| Within 65+ (Pneumococcal) | NA | NA | NA | NA | **1.06 (1.05-1.07)** | **1.06 (1.05-1.07)** | NA | NA | NA | NA | NA | NA |
| **Marital Status** |  |  |  |  |  |  |  |  |  |  |  |  |
| Married | ref | ref | ref | ref | ref | ref | ref | ref | ref | ref | ref | ref |
| Not married | **1.75 (1.53-2.00)** | **1.72 (1.51-1.97)** | **0.98 (0.67-0.73)** | 0.96 (0.91-1.00) | **0.89 (0.80-0.99)** | **0.88 (0.79-0.97)** | **0.88 (0.84-0.92)** | **0.87 (0.84-0.92)** | 0.97 (0.92-1.01) | **0.94 (0.90-0.99)** | **0.87 (0.80-0.95)** | **0.87 (0.80-0.95)** |
| **Education Level** |  |  |  |  |  |  |  |  |  |  |  |  |
| Bachelor's or higher | ref | ref | ref | ref | ref | ref | ref | ref | ref | ref | ref | ref |
| HS graduate/GED/some college | **0.49 (0.43-0.55)** | **0.47 (0.41-0.54)** | **0.71 (0.68-0.74)** | **0.71 (0.67-0.74)** | 0.91 (0.81-1.03) | 0.91 (0.81-1.02) | **0.66 (0.63-0.69)** | **0.65 (0.62-0.68)** | **0.89 (0.86-0.94)** | **0.90 (0.86-0.94)** | **0.73 (0.68-0.79)** | **0.73 (0.68-0.79)** |
| Less than high school | **0.30 (0.22-0.42)** | **0.29 (0.21-0.41)** | **0.43 (0.39-0.48)** | **0.42 (0.38-0.48)** | **0.67 (0.56-0.79)** | **0.67 (0.56-0.80)** | **0.61 (0.55-0.66)** | **0.58 (0.53-0.63)** | **0.74 (0.68-0.81)** | **0.75 (0.69-0.83)** | **0.45 (0.39-0.53)** | **0.46 (0.39-0.54)** |
| **Family income** |  |  |  |  |  |  |  |  |  |  |  |  |
| >$75000 | ref | ref | ref | ref | ref | ref | ref | ref | ref | ref | ref | ref |
| $35000-$74999 | **0.75 (0.65-0.87)** | **0.77 (0.67-0.89)** | 0.95 (0.90-1.00) | 0.96 (0.91-1.02) | 0.94 (0.82-1.06) | 0.93 (0.82-1.06) | **0.82 (0.78-0.87)** | **0.83 (0.79-0.87)** | **0.92 (0.87-0.97)** | **0.92 (0.87-0.97)** | **0.88 (0.80-0.96)** | **0.87 (0.79-0.96)** |
| <$35000 | **0.84 (0.71-1.00)** | **0.83 (0.70-0.99)*** | 0.96 (0.89-1.03) | 0.97 (0.91-1.05) | **0.84 (0.72-0.97)** | **0.84 (0.72-0.97)** | **0.84 (0.79-0.90)** | **0.85 (0.79-0.91)** | **0.82 (0.77-0.88)** | **0.84 (0.79-0.90)** | **0.66 (0.59-0.74)** | **0.65 (0.58-0.73)** |
| **Health insurance status** |  |  |  |  |  |  |  |  |  |  |  |  |
| Private only | ref | ref | ref | ref | ref | ref | ref | ref | ref | ref | ref | ref |
| Public only | 0.88 (0.75-1.05) | 0.92 (0.77-1.09) | 0.93 (0.86-1.00) | 0.94 (0.87 -1.01) | **0.74 (0.63-0.87)** | **0.75 (0.64-0.88)** | **0.84 (0.79-0.90)** | **0.84 (0.79-0.90)** | **0.91 (0.85-0.97)** | **0.90 (0.84-0.97)** | 0.90 (0.80-1.02) | 0.91 (0.80-1.04) |
| Both private and public | 0.97 (0.53-1.86) | 0.96 (0.52-1.77) | 1.20 (0.92-1.58) | 1.14 (0.87-1.49) | 0.96 (0.59-1.55) | 0.91 (0.57-1.45) | 1.21 (0.93-1.57) | 1.26 (0.97-1.64) | 0.97 (0.75-1.27) | 0.94 (0.73-1.22) | 1.27 (0.86-1.87) | 1.20 (0.82-1.77) |
| Not covered | 0.84 (0.67-1.06) | 0.86 (0.68-1.08) | **0.80 (0.73-0.88)** | **0.81 (0.74-0.89)** | **0.14 (0.07-0.30)** | **0.10 (0.04-0.27)** | **0.36 (0.33-0.40)** | **0.35 (0.31-0.39)** | **0.76 (0.70-0.82)** | **0.76 (0.70-0.82)** | **0.24 (0.16-0.36)** | **0.24 (0.16-0.36)** |
| **Self-reported health status** |  |  |  |  |  |  |  |  |  |  |  |  |
| Excellent/very good | ref | ref | ref | ref | ref | ref | ref | ref | ref | ref | ref | ref |
| good | 0.93 (0.80-1.07) | 0.94 (0.82-1.09) | 0.95 (0.86-1.00) | **0.94 (0.89-0.99)** | **1.12 (1.01-1.25)** | 1.11 (0.99-1.24) | 1.03 (0.99-1.09) | **1.05 (1.00-1.10)** | **0.93 (0.88-0.97)** | **0.92 (0.88-0.97)** | **0.76 (0.70-0.83)** | **0.77 (0.71-0.84)** |
| fair/poor | 0.84 (0.64-1.12) | 0.86 (0.65-1.14) | **0.87 (0.81-0.95)** | **0.86 (0.79-0.94)** | 1.09 (0.04-1.25) | **1.12 (0.97-1.29)** | **1.09 (1.01-1.17)** | **1.10 (1.02-1.19)** | **0.84 (0.78-0.91)** | **0.85 (0.78-0.92)** | **0.64 (0.57-0.72)** | **0.65 (0.59-0.77)** |
| **Number of visits to doctor's office in past 12 months** |  |  |  |  |  |  |  |  |  |  |  |  |
| 8 visits or more | ref | ref | ref | ref | ref | ref | ref | ref | ref | ref | ref | ref |
| 4-7 visits | 0.96 (0.81-1.13) | 0.96 (0.81-1.14) | **0.87 (0.81-0.94)** | **0.87 (0.81-0.93)** | **0.78 (0.68-0.89)** | **0.77 (0.68-0.88)** | **0.86 (0.81-0.91)** | **0.29 (0.27-0.31)** | **0.84 (0.78-0.90)** | **0.83 (0.78-0.89)** | **1.01 (0.92-1.11)** | **1.01 (0.92-1.11)** |
| 2-3 visits | **0.82-(0.71-0.96)** | **0.85 (0.73-0.99)** | **0.83 (0.78-0.89)** | **0.84 (0.79-0.90)** | **0.65 (0.57-0.74)** | **0.65 (0.56-0.74)** | **0.70 (0.66-0.74)** | **0.70 (0.66-0.74)** | **0.75 (0.70-0.80)** | **0.75 (0.70-0.80)** | **0.84 (0.76-0.92)** | **0.85 (0.77-0.93)** |
| 1 visit | **0.64 (0.53-0.78)** | **0.67 (0.55-0.82)** | **0.74 (0.69-0.80)** | **0.74 (0.69-0.80)** | **0.48 (0.40-0.56)** | **0.48 (0.41-0.57)** | **0.51 (0.48-0.55)** | **0.51 (0.48-0.55)** | **0.60 (0.55-0.64)** | **0.59 (0.55-0.64)** | **0.67 (0.59-0.72)** | **0.68 (0.59-0.77)** |
| No visit | **0.51 (0.41-**  **0.64)** | **0.53 (0.42-0.67)** | **0.60 (0.55-0.64)** | **0.62 (0.57-0.67)** | **0.20 (0.16-0.24)** | **0.19 (0.15-0.23)** | **0.28 (0.26-0.31)** | **0.29 (0.27-0.31)** | **0.40 (0.37-0.43)** | **0.40 (0.37-0.53)** | **0.30 (0.25-0.37)** | **0.31 (0.25-0.37)** |

Note: Boldface indicates statistically significant (*p*<0.05) difference in odds ratio from the reference level by Wald test.

Note: The estimated unadjusted Pneumonia vaccination rate for US born Asian Indians (*) has extremely large odds ratio due to small sample size (only 4 Asian Indians in the sample are US born, over 65 years of age, and indicated whether they have received the Pneumonia vaccine.

Note: The estimated unadjusted Shingles vaccination rate for US born Asian Indians (*) has extremely large odds ratio due to small sample size (only 5 Asian Indians in the sample are US born, over 60 years of age, and indicated whether they have received the Shingles vaccine.

^a^Adjusted odds ratios, adjusted for all variables included in the above table.
